# Comparative Effectiveness of Anti-Inflammatory Drug Treatments in Coronary Heart Disease Patients: A Systematic Review and Network Meta-Analysis

**DOI:** 10.1155/2021/5160728

**Published:** 2021-01-14

**Authors:** Ivan Wudexi, Elica Shokri, Mohamed Abo-Aly, Kazuhiro Shindo, Ahmed Abdel-Latif

**Affiliations:** Gill Heart Institute and Division of Cardiovascular Medicine, University of Kentucky and the Lexington VA Medical Center, Lexington, KY, USA

## Abstract

**Methods:**

We conducted a network meta-analysis of randomized controlled trials that studied the effects of anti-inflammatory medications on cardiovascular outcomes of coronary artery disease patients. We searched the electronic database until March 2020 for relevant studies.

**Results:**

Nineteen trials examining the efficacy of eight anti-inflammatory medications (pexelizumab, anakinra, colchicine, darapladib, varespladib, canakinumab, inclacumab, and losmapimod) were selected for analysis. Overall, there is no statistically significant difference in all-cause mortality, cardiovascular mortality, revascularization, and major cardio and cerebrovascular events (MACCE) with the use of anti-inflammatory drugs. However, we found the use of colchicine significantly reduces the odds of developing stroke by approximately 75% (OR 0.26, CI 0.10-0.63). Colchicine use was also associated with a lower risk of revascularization and MACCE compared to the other agents. Our subgroup analyses comparing the timing of medication initiation (within 7 days vs. >7 days) and clinical presentation (ACS vs. non-ACS) revealed a significant reduction in the risk of recurrent MI in the group that received medication after seven days (OR 0.92, CI 0.86-0.99) and the non-ACS group (OR 0.88, CI 0.80-0.98).

**Conclusion:**

Although many anti-inflammatory medications have failed to reduce adverse cardiovascular outcomes in the CAD population, selected medications show promise among subgroups of patients without ACS or after the first week following an acute ischemic event. Future studies examining the proper timing and targetable anti-inflammatory pathways are warranted.

## 1. Introduction

Each year, roughly 1.1 million patients are hospitalized with an acute coronary syndrome (ACS) such as an acute myocardial infarction (AMI) event in the United States [1]. Despite the use of optimal guideline, directed medical therapies and secondary prevention, recurrent ischemic coronary events, and mortality remain high among patients with coronary artery disease, with an annual rate of 4 to 5% after their initial ischemic event. Accordingly, ischemic heart disease remains the leading cause of heart failure and mortality in the western world. Over the past 2 decades, there has been an increasing interest in discovering therapeutic agents for reducing residual risk among patients with acute coronary syndromes (ACS), including ST segment elevation myocardial infarction (STEMI), non-ST segment myocardial infarction (NSTEMI), and unstable angina [2, 3].

Decades of animal and human research have confirmed the critical role that inflammation plays in the development and progress of atherosclerosis. Innate immune cells such as neutrophils and proinflammatory monocytes play a critical role in the body's response to sterile tissue injury such as after myocardial infarction [4]. While the immune response is necessary for clearing dead cells and preparing the myocardium for healing, exacerbated inflammation, as often seen in mammals, can lead to detrimental effects. Studies have shown strong correlation between elevated neutrophil and monocyte count and infarction expansion as well as poor clinical outcomes after AMI [5–8]. Furthermore, attempts at modulating the immune response after AMI have resulted in attenuated myocardial damage, reduced atherosclerosis burden, and enhanced survival [9–11]. This increased interest in targeting inflammation using clinically relevant therapies.

Modulating the inflammatory response post-AMI is an elusive target as studies have shown that complete systemic suppression of inflammation is rather harmful [12]. Delayed healing and ventricular aneurysms were reported with glucocorticosteroids [13]. Similarly, nonsteroidal anti-inflammatory drug use in coronary artery disease (CAD) patients is associated with higher mortality and recurrent AMI [14]. Additionally, studies aimed at depleting inflammatory cells failed to demonstrate benefit [15]. On the other hand, selective targeting of inflammatory mediators such as IL-1*β* using selective monoclonal antibodies demonstrated success in clinical studies, albeit with high cost and modest benefit [16–18]. Therefore, strategies aimed at modulating the inflammatory response rather than its suppression provide therapeutic promise. However, studies using selective targets of the inflammatory pathways in CAD patients remain small and underpowered to reach conclusions. Furthermore, with multiple new targeted anti-inflammatory agents (referred to as anti-inflammatory drugs throughout the manuscript), agents being studies in the field, there are no head-to-head comparisons between and conducting such a comparison using the randomized design will be prohibitively expensive. Studies on human subjects were limited to a small study sample and no head-to-head comparisons [19]. In this meta-analysis, we seek to perform a cumulative analysis of the efficacy of new anti-inflammatory drugs to reduce clinical events among CAD patients. We also performed a network meta-analysis to investigate the efficacy and safety between these agents.

## 2. Materials and Methods

We conducted this protocol-driven systematic review and meta-analysis according to the Preferred Reported Items for Systematic Reviews and Meta-Analyses (PRISMA) [20]. We systematically searched PubMed, Cochrane, and Scopus for relevant studies through March 2020. The search formula for each database is provided in detail in the online supplementary appendix (available here). We also screened the references of relevant meta-analysis and systematic reviews for eligible studies. The abstracts of the American Heart Association, American College of Cardiology, and European Society of Cardiology were screened over the last 2 years for eligible studies.

We included randomized controlled trials (RCTs) that studied the effects of the following anti-inflammatory medications on cardiovascular outcomes of patients with coronary artery disease: pexelizumab, colchicine, darapladib, varespladib, anakinra, canakinumab, inclacumab, and losmapimod. Our primary cardiovascular outcomes of interest were all-cause death, cardiovascular death, recurrent myocardial infarction, stroke, revascularization, and major adverse cardiac and cerebrovascular events (MACCE), defined as a composite of death/cardiovascular death, myocardial infarction, and stroke. In studies that did not report this definition of MACCE, we accepted the closest composite to our MACCE definition. In studies that use multiple dosages of a medication, we use the highest dose except when data is only available for a lower dose across multiple studies of the same medication. In studies that reported clinical outcomes at multiple follow-up lengths, we included the data from the longest available follow-up duration. We excluded studies that did not have the cardiovascular outcomes of interest or enrolled patients with significant medical comorbidities that may affect the outcomes (e.g., cancer patients who receive chemotherapy).

Two reviewers (E.S and I.W) independently screened and evaluated the eligibility of the studies using the aforementioned criteria. Studies were initially screened by title and abstract. After initial screening, the full text of the identified studies was reviewed thoroughly to determine their eligibility. Baseline characteristics and clinical outcomes of interest were extracted using a standardized data extraction form. Disagreements were resolved through discussions, and the opinion of a third investigator (M.A.) was requested if necessary. Quality assessment of the studies was performed using the Cochrane Collaboration's risk of bias tool [21].

### 2.1. Statistical Analyses

The prespecified outcomes of our analyses were all-cause mortality, cardiac mortality, recurrent myocardial infarction, stroke, revascularization, and major adverse cardiac and cerebrovascular events (MACCE). Summary estimates were calculated as odds ratios (OR) with 95% confidence intervals (CI) using the random-effects model based on DerSimonian and Laird's meta-analytic statistical method [22]. Considering the heterogeneity of the included trials and its potential influence on treatment effects, we prespecified the use of random-effects model to assess effect sizes. The *I*^2^ index was used to summarize the proportion of total variability in the estimate. The *I*^2^ statistic is derived from the *Q* statistic and describes the percentage of total variation across studies attributed to heterogeneity; values of 25%, 50%, and 75% correspond to low, moderate, and high heterogeneity, respectively [23, 24]. We performed subgroup analyses according to (a) type of enrolled population in studies (stable coronary heart disease and CABG vs. ACS) and (b) the duration between symptom onset/index event and study medication administration. We used visual inspection of funnel plots to assess for publication bias. The statistical level of significance was 2-tailed *P* < 0.05. Analyses were performed using Review Manager version 5.3 (Revman; The Cochrane Collaboration, Oxford, UK).

In the network meta-analysis, we used the Bayesian Markov chain Monte Carlo modelling using the informative prior setting. The Bayesian model was selected because of its greater flexibility and ability to rank treatments according to their comparative effectiveness. Informative prior was chosen to assume consistency between heterogeneity variances and ensure a realistic heterogeneity estimation [25]. All network analyses were performed with NetMetaXL 1.6.1 (Canadian Agency for Drugs and Technologies in Health, Ottawa, Canada) and WinBUGS 1.4.3 (MRC Biostatistics Unit, Cambridge, United Kingdom) [26]. A burn-in phase of 20,000 iterations was used to achieve convergence. The convergence was assessed using the Brooks-Gelman-Rubin plots. NetMetaXL fits three chains for Bayesian network meta-analysis. We evaluated heterogeneity using the between-study heterogeneity variances per outcome, known as *τ*^2^ (tau-squared) and its 95% CI. Inconsistency was evaluated by plotting the posterior mean deviation of individual data points in the inconsistency model against its posterior mean deviation in the consistency model to identify any loop in the treatment network where inconsistency exists [27].

## 3. Results

**Figure 1:**
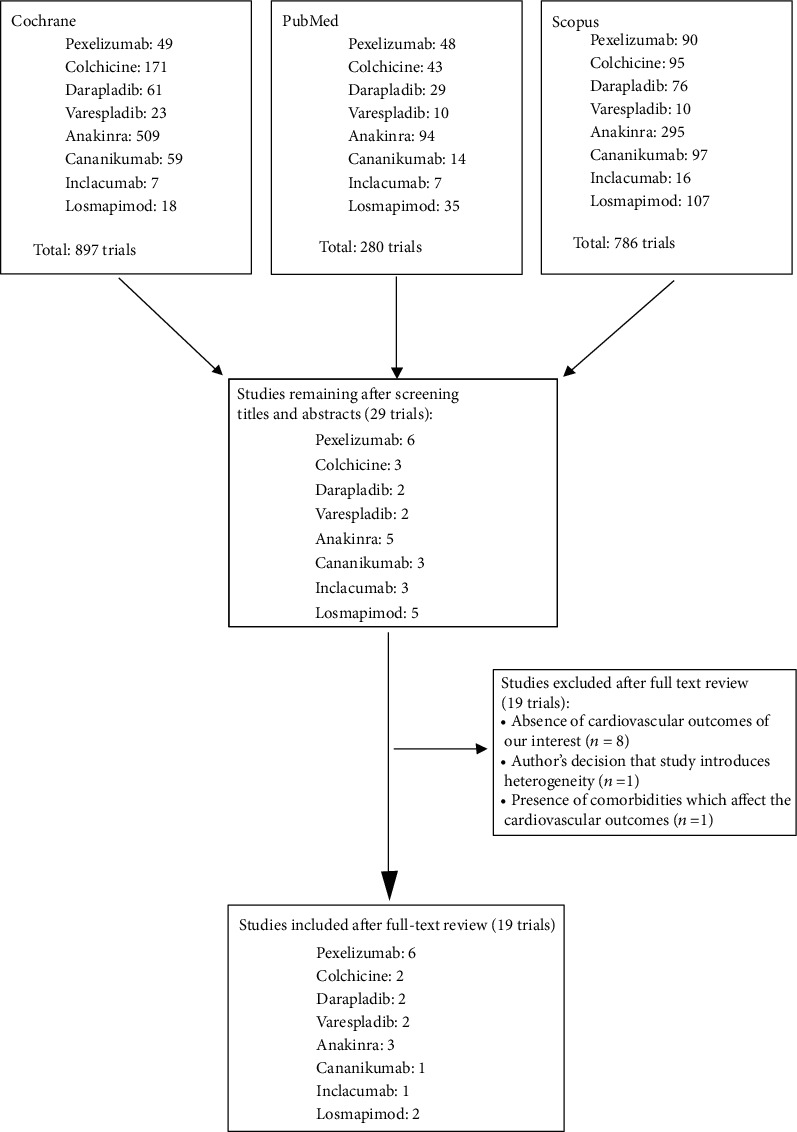
Flow chart showing the search algorithm and final study selection in the meta-analysis.

Our literature search yielded a total of 1963 trials, as shown in Figure 1. After title and abstract screening and full-text review, nineteen trials including 70,620 patients met our inclusion criteria utilizing the following medications: pexelizumab (6 studies) [16, 28–32], anakinra (3 studies) [33–35], colchicine (2 studies) [36, 37], darapladib (2 studies) [38, 39], varespladib (2 studies) [40, 41], canakinumab (1 study) [17], inclacumab (1 study) [42], and losmapimod (2 studies) [3, 43]. The baseline characteristics of the study population are shown in Table 1. The median patient age ranges from 53-67 years old, 77.3% of the patient population were males, and the follow-up duration ranged from 30 days to 3.7 years. Overall, patients included in this meta-analysis had the typical comorbidities of this cohort including diabetes, hypertension, hyperlipidemia, and high prevalence of smoking. Population of patients included in our meta-analysis ranged from chronic CAD (34.8%), patients undergoing CABG (11.7%), and ACS patients (57%). Of the total number of 19 studies, 6 trials enrolled stable coronary artery disease [30–32, 36, 39, 42] patients, 3 trials investigated ACS patients with spectrum of unstable angina to STEMI [38, 40, 41], 3 trials enrolled patients with either STEMI or NSTEMI [17, 37, 43], 5 studies only included patients who presented with STEMI [16, 28, 29, 33, 34], and 2 trials enrolled only NSTEMI patients [3, 35].

The breakdown of the trials with the corresponding patient populations is shown in Table 2. The quality assessment of the included studies is displayed in Supplemental Table 1 of the Appendix.

### 3.1. Meta-Analysis Results

**Figure 2:**
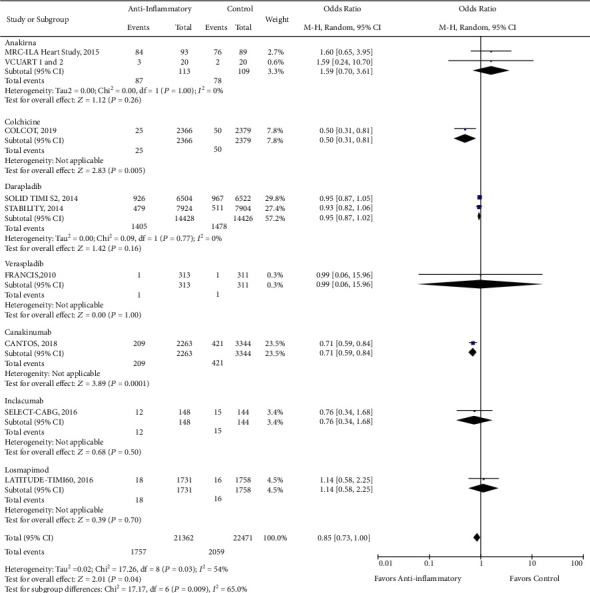
Forest plot for the comparative risk of revascularization with anti-inflammatory therapy versus standard of care alone. Anti-inflammatory therapy significantly reduced the risk of revascularization (OR 0.85, CI 0.73-1.00; *P* < 0.05).

**Figure 3:**
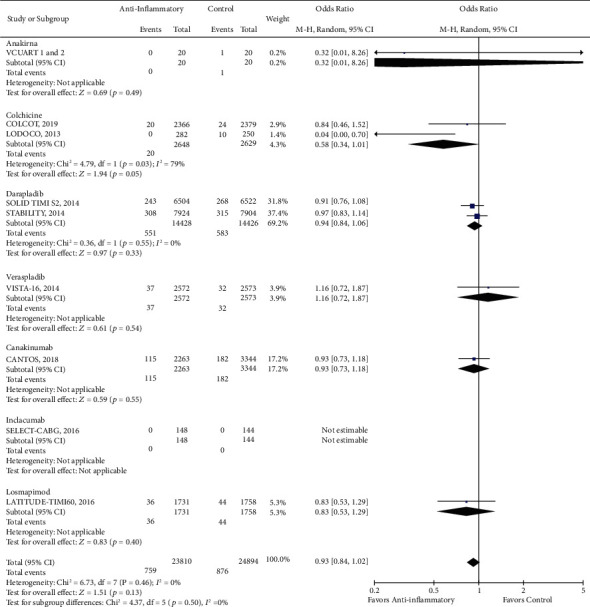
Forest plot of cardiac mortality. There was no significant difference between anti-inflammatory therapy and standard therapy alone regarding cardiovascular mortality (OR 0.93, CI 0.84-1.02; *P* = 0.13).

**Figure 4:**
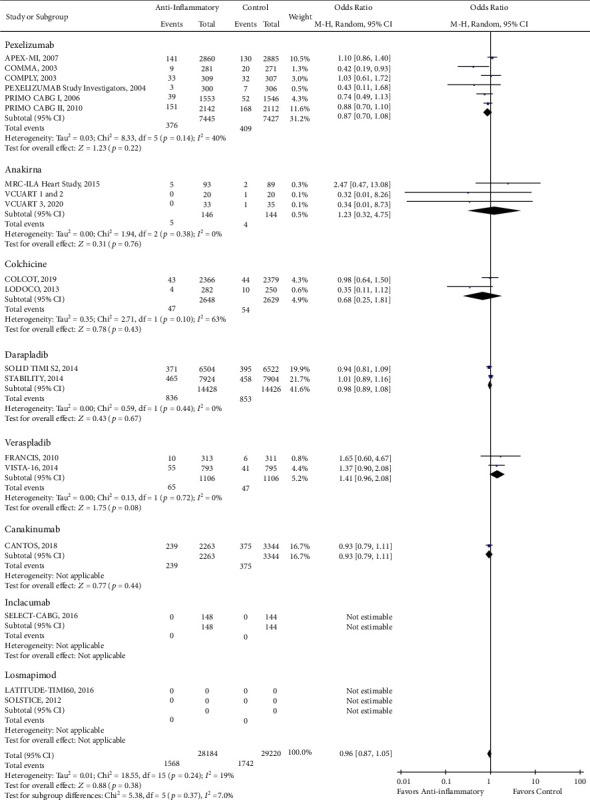
Forest plot of all-cause mortality. No significant difference was observed in the risk of all-cause mortality between anti-inflammatory therapy and standard therapy alone (OR 0.96, CI 0.87-1.05; *P* = 0.38).

**Figure 5:**
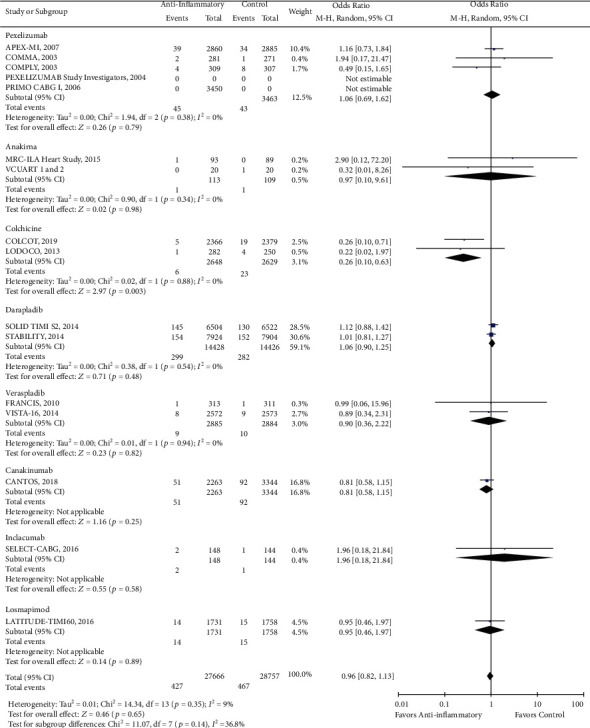
Forest plot of stroke. There was no significant difference between anti-inflammatory therapy and standard therapy alone in regard to stroke (OR 0.96, CI 0.82-1.13; *P* = 0.65).

**Figure 6:**
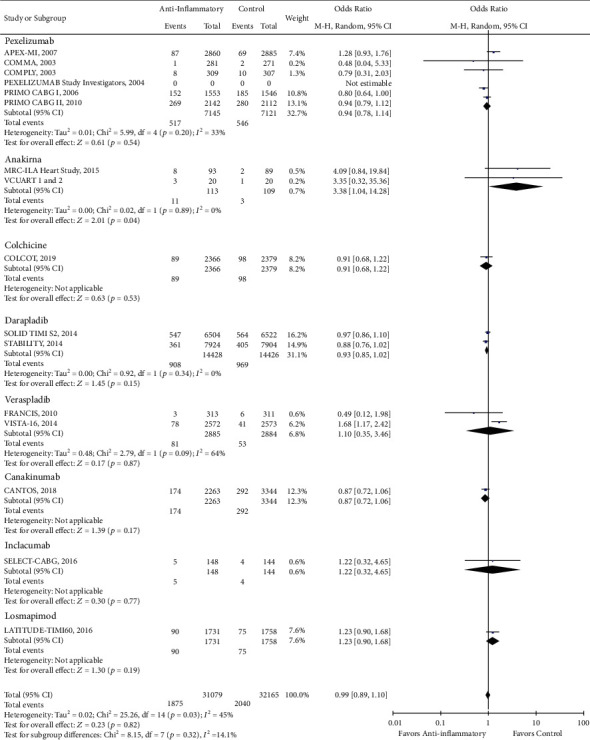
Forest plot of recurrent myocardial infarction. No significant difference was observed between anti-inflammatory therapy and standard therapy in terms of recurrent myocardial infarction (OR 0.99, CI 0.89-1.10; *P* = 0.82).

**Figure 7:**
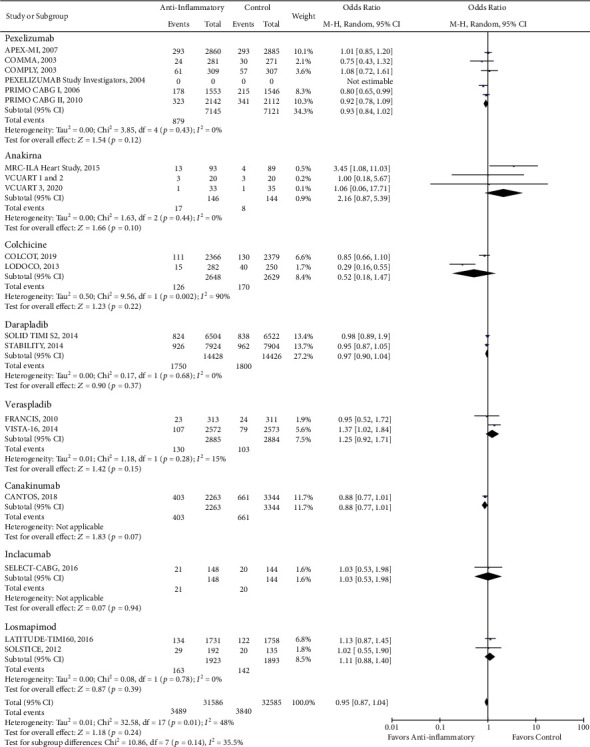
Forest plot of MACCE. There was no significant difference between anti-inflammatory therapy and standard therapy alone regarding MACCE (OR 0.95, CI 0.87-1.04; *P* = 0.24).

There was a reduction in the risk of revascularization (OR 0.85, CI 0.73-1.00; *P* = 0.04) with the use of anti-inflammatory drugs compared to standard of care alone (Figure 2). However, there was no statistically significant difference in cardiovascular mortality (OR 0.93, CI 0.84-1.02; *P* = 0.13), all-cause mortality (OR 0.96, CI 0.87-1.05; *P* = 0.38), stroke (OR 0.96, CI 0.82-1.13; *P* = 0.65), recurrent myocardial infarction (OR 0.99, CI 0.89-1.10; *P* = 0.82), and major adverse cardio and cerebrovascular events (MACCE) (OR 0.95, CI 0.87-1.04; *P* = 0.24) with the use of anti-inflammatory medications (Figures 3–7).

While we did not observe a significant reduction in the incidence of stroke with the use of anti-inflammatory drugs, we found the use of colchicine significantly reduces the stroke odds by approximately 75% in patients with coronary artery disease (OR 0.26, CI 0.10-0.63; *P* = 0.003) (Figure 5). On the other hand, we found that the use of anakinra was associated with an almost fourfold increase in the risk of developing recurrent MI (OR 3.85, CI 1.04-14.28; *P* = 0.04) (Figure 6). Lastly, although the PRIMO CABG trial had previously shown that pexelizumab treatment improves MACCE in patients undergoing CABG, our pooled analysis showed only a trend in the reduction of MACCE with pexelizumab compared to placebo (OR 0.93, CI 0.84-1.02; *P* = 0.12) (Figure 7).

### 3.2. Subgroup Analysis

#### 3.2.1. Acute vs. Subacute vs. Chronic Presentation

The results of the subgroup analyses according to time of study medication initiation after symptom onset/index event (7 days vs. >7 days) are summarized in Table 3. Patients who were given study medications after 7 days of index ischemic event demonstrated reduced risk for developing recurrent MI and revascularization (*P* value for interaction = 0.03). There was also a trend in favor of drug initiation after 7 days from symptom onset or index clinical event in all-cause mortality, cardiac mortality, and MACCE.

### 3.3. ACS vs. Non-ACS Presentation

The sensitivity analysis based on clinical presentation (ACS vs. non-ACS) is summarized in Table 3. In our study, the use of anti-inflammatory medications was found to reduce the risk of recurrent MI in the non-ACS population compared to the ACS population (OR 0.88, CI 0.80-0.98; *P* = 0.04). There was also a trend in favor of starting anti-inflammatory drugs in non-ACS patients when MACCE was assessed. Meanwhile, no significant differences in other cardiovascular outcomes (e.g., all-cause mortality, CV death, stroke, and recurrent MI) were noted in our analyses.

### 3.4. Network Meta-Analysis

Supplemental Figures 1–6 show the forest plot comparing the relative efficacies of each anti-inflammatory medication on all-cause mortality, cardiovascular death, recurrent myocardial infarction, revascularization, stroke, and MACCE. We found the use of colchicine was significantly associated with reduced risk of revascularization, stroke, and MACCE in comparison with several anti-inflammatory medications in our study. Specifically, colchicine was associated with lower risk of revascularization events than both anakinra and darapladib (OR 0.31, CI 0.11-0.84 and OR 0.52, CI 0.29-0.93). It was also associated with lower risk of stroke after MI when compared with the use of darapladib, pexelizumab, losmapimod, canakinumab, and varespladib (OR 0.23, CI 0.07-0.57; OR 0.23, CI 0.07-0.64; OR 0.25, CI 0.07-0.85; OR 0.30, CI 0.09-0.81; and OR 0.26, CI 0.07-0.97, respectively). Furthermore, colchicine use was also associated with lower risk of MACCE compared to darapladib, losmapimod, anakinra, or varespladib (OR 0.69, CI 0.44-0.98; OR 0.60, CI 0.37-0.93; OR 0.28, CI 0.10-0.70; and OR 0.53, CI 0.32-0.83, respectively). Visualization of the network meta-analysis is depicted in Supplemental Figure 7.

### 3.5. Assessment of Heterogeneity

We drew funnel plots to seek evidence of publication bias: where inconsistency was high, the funnel plots were not interpretable; where inconsistency was low, the funnel plots were inconclusive (Supplemental Figure 8). In the network meta-analysis, we did not observe significant inconsistency in our analysis of the outcomes (Supplemental Figure 9).

## 4. Discussion

The use of selective anti-inflammatory drugs to reduce the incidence of cardiovascular events in high-risk patients with CAD is debatable in the clinical community. We conducted a systematic review and network meta-analysis to study the effect of several anti-inflammatory medications on cardiovascular outcomes in coronary artery disease patients. In our systematic review of eight anti-inflammatory medications, we observed a modest reduction in cardiovascular outcomes when compared with placebo. However, as previously published, the use of colchicine and canakinumab did show a beneficial effect on reducing the risk of revascularization post-AMI [17, 37]. Furthermore, there was a significant reduction in the incidence of stroke in the CAD population who received colchicine as compared with the placebo-treated group. Colchicine use was also associated with lower odds of MACCE compared to other selective anti-inflammatory agents.

Our subgroup analyses based on the timing of medication initiation (<7 days vs. >7 days) and patient's clinical presentation (ACS vs. non-ACS) demonstrated interesting and unexpected results. Theoretically, the administration of anti-inflammatory agents is expected to demonstrate benefit during heightened inflammation after ACS. However, our subgroup analyses suggest that anti-inflammatory drugs could potentially be more beneficial among patients with non-ACS presentation and those initiated on therapy after 7 days from symptom onset. This could be explained by the physiological importance of the nonsterile inflammatory response early after coronary ischemia [12]. It has been demonstrated in animal studies that depleting inflammatory cells such as neutrophils and macrophages after AMI is rather detrimental and could result in increased mortality and exacerbation of heart failure. To our knowledge, there has not been a human study that directly compares the efficacy of anti-inflammatory medications given at different time points after index events. We believe future studies that compare the timing of medication administration and its association with cardiovascular outcomes are necessary to further explore the underpinnings of this phenomenon.

### 4.1. Colchicine and Stroke Incidence

Our study finding was consistent with a recently published meta-analysis study that looked into the efficacy of colchicine in preventing stroke in patients with coronary artery disease. Even though our analysis excluded two studies included in this meta-analysis, due to the absence of cardiovascular outcomes as either primary or secondary endpoints, a similar degree of stroke risk reduction was demonstrated (OR 0.31, 95% confidence interval: 0.13-0.71; *P* = 0.006) [44]. The proposed mechanisms of action of colchicine are through its effect on neutrophil and monocytes through inhibition of NLRP3 inflammasome complex formation and microtubule function, which inhibits the production of IL-1 beta and IL-18 and prevents the migration of inflammatory cells, respectively. Inhibition of these pathways had been shown to decrease both hsCRP levels and atherosclerotic plaque progression as well as instability on CT scan [45]. It is worth noting that the pooled clinical benefit seen with the use of colchicine in our study largely derived from the study population with acute myocardial infarction as opposed to stable coronary disease [36, 37]. It is possible that this finding is related to higher transcoronary gradients of IL-1 and IL-18 seen in the ACS than the stable CAD population [46]. Thus, inhibition of these interleukins by colchicine might explain the reduced risk of stroke in patients with recent MI, as had been demonstrated in previous RCT [37].

### 4.2. Anakinra and Recurrent MI

Our findings are consistent with a recent meta-analysis that studied the effect of IL-1 blockage on cardiovascular risk. The authors found an overall increased risk of recurrent MI with anakinra after pooling data from a total of five anakinra trials [47]. Although we only included three of the five trials based on our prespecified inclusion criteria (we excluded one study that included only the heart failure population and another study that did not have cardiovascular outcome as either primary or secondary end points) in our analysis, we found similar risk estimates with a wide confidence interval. This finding is in contrast with the CANTOS trial where the use of another IL-1 blockade medication, canakinumab, had resulted in decreased risk of recurrent MI and revascularization events after AMI [17]. Although no definite explanation exists yet as per our knowledge, we believe it is possible that the timing and duration of drug administration may play a crucial role in determining response to medication.

### 4.3. Pexelizumab and Cardiovascular Outcomes

Our finding was consistent with a previous meta-analysis that included seven pexelizumab studies. They also did not find any significant improvements in major adverse cardiovascular outcomes and its components with the use of pexelizumab compared with placebo. However, they did find a 26% decreased risk of death in the CABG subpopulation (OR 0.74 [0.58–0.94]; *P* = 0.01). It is hypothesized that the benefit seen in primarily CABG population may be related to the presence of intact microvascular system, salvageable myocardium, ability of the medication to penetrate the tissues, differences in the inflammatory pathway involved, and degree of complement system activation in this population as compared to the ACS population. Upstream delivery of pexelizumab in the STEMI population may not be effective as irreversible damage to the vascular system and myocardium prevents the penetration of medication to the site of inflammation yielding the drug less effective [48].

## 5. Study Limitations

There were some limitations to this study. First, although we included all patients with coronary artery disease, they range in severity of clinical presentation from stable coronary heart disease to acute coronary syndrome requiring revascularization procedure (PCI or CABG). This heterogeneity could explain some of our findings and we aimed to address them by conducting extensive subgroup analyses. Second, there is variability in the length of follow-up among trials from 30 days to 3.7 years. Third, the three anakinra trials that were included in our analysis are small studies with less than 200 patients. Although we see a potential harm with the use of anakinra (increased risk of recurrent MI), the potential bias with the small study sample must not be overlooked. Further larger anakinra studies will be required to explore this interesting association. Fourth, the trials of two medications in our study (pexelizumab and varespladib) did not exactly have the same intervention protocol. Two out of the six pexelizumab trials (COMMA and COMPLY) were given an infusion of only 20 hours as opposed to the 24-hour duration studies in the rest of the studies. Additionally, although both varespladib trials (FRANCIS and VISTA-16 trials) utilized the same dose of varespladib, the duration of treatment and the statin dose are different (24 vs. 16 weeks and 80 mg vs. at least 20 mg, respectively).

Despite these limitations, our analysis has several strengths. To our knowledge, this is the first study to assess the cumulative efficacy of eight different anti-inflammatory medications and compare their individual efficacy on cardiovascular outcomes of patients with coronary artery disease. Second, our study was also the first to analyze the efficacy of anti-inflammatory medication based on timing of drug administration and patient's clinical presentation. Third, our outcomes of interest (e.g., death, CV death, and MI) are largely objective findings and most of the trials included in our study have independent event adjudicators, thus minimizing the risk of measurement bias.

## 6. Conclusion

When applied to a largely unselected patient population with coronary artery disease, anti-inflammatory medications failed to reduce adverse cardiovascular outcomes. However, selected agents show promise among subgroups of patients without ACS or after the first week following an acute ischemic event. Future studies examining the proper timing and targetable inflammatory pathways are warranted.

## Figures and Tables

**Table 1:** Characteristics of included studies.

Trials	Study design	Year of study	No. of patients	Study population	Lost to F/U	Age	Female %	Smoking history	DM	HTN	HLD	Prior ACS/MI	Prior CHF	Prior PCI	Prior CABG	CRP (mg/L)	IL-6 (mg/L)
APEX AMI [28]	RCT	2007	2860 (T)	STEMI (100)	6 (0.2)	61 (51-71)	691 (24.2)	1226 (42.9)	446 (15.6)			340 (11.9)	105 (3.7)	278 (9.7)	60 (2.1)		
			2885 (P)	STEMI (100)	9 (0.3)	61 (52-71)	634 (22.0)	1252 (43.6)	467 (16.2)			354 (12.3)	103 (3.6)	284 (9.9)	68 (2.4)		
COMMA [16]	RCT	2003	262 (T)	STEMI (100)	3 (1.1)	58 (50 to 72)	24%	37%	20%	64%		16%	9%				
			242 (P)	STEMI (100)	4 (1.7)	61 (51 to 70)	24%	40%	21%	61%		17%	9%				
COMPLY [16]	RCT	2003	309 (T)	STEMI (100)	0	62 (52 to 70)	28%	40%	14%	59%		22%	13%				
			307 (P)	STEMI (100)	1 (0.3)	60 (52 to 70)	33%	42%	16%	56%		17%	8.50%				
PRIMO CABG [32]	RCT	2006	1553 (T)	CABG w/wo concurrent valve surgery (100)	7	65.1 (10.2)^∗^	440 (28.3)		376 (24.2)	1103 (71.0)		599 (38.6)	245 (15.8)	101 (6.5)	133 (8.6)		
			1546 (P)	CABG w/wo concurrent valve surgery (100)	10	65.3 (10.3)^∗^	391 (25.3)		373 (24.1)	1104 (71.4)		624 (40.4)	219 (14.2)	109 (7.1)	128 (8.3)		
PRIMO CABG II [31]	RCT	2010	2142 (T)	CABG w/wo concurrent valve surgery (100)	18 (0.8)	66.2 (31–91) ^∗^	839 (39.2)		1298 (60.6)				838 (39.1)		228 (10.6)		
			2112 (P)	CABG w/wo concurrent valve surgery (100)	19 (0.9)	66.2 (35–90)^∗^	852 (40.3)		1246 (59.0)				843 (40.0)		199 (9.4)		
Pexelizumab study investigators [30]	RCT	2004	300 (T)	CABG w/wo concurrent valve surgery (100)	NA												
			306 (P)	CABG w/wo concurrent valve surgery (100)	NA												
MRC-ILA Heart Study [35]	RCT	2015	98 (T)	NSTEMI (100)	2(2.0)	61.4 (11.7)^∗^	35 (32.3)	34 (36.6)	15 (15)	31 (33.3)	27 (29.0)	23 (24.7)				5.38 (4.12, 7.04)	6.01 (4.68, 7.72)
			89 (P)	NSTEMI (100)	0	61.3 (12.3)^∗^	22(34.7)	31 (34.8)	8 (9)	29 (32.6)	28 (31.5)	24 (27.0)				5.21 (3.75, 7.22)	5.23 (3.98, 6.88)
VCU-ART 1&2 [33]	RCT	2015	20 (T)	STEMI (100)	0	57 (48-60)	8 (40)	12 (60)	5 (25)	12 (60)	14 (70)						
			20 (P)	STEMI (100)	0	58 (51-65)	2 (10)	14 (70)	4 (20)	14 (70)	13 (65)						
VCU-ART 3 [34]	RCT	2020	33 (T)	STEMI (100)	0	53 (49-62)	9 (27)		6 (18)	13 (39)							
			35 (P)	STEMI (100)	0	56 (51-65)	5 (14)		15 (43)	23 (66)							
LODOCO [36]	RCT	2010	282 (T)	Stable Coronary Heart Disease (100)	0	66 ± 9.6	31 (11)	10 (4)	92 (33)			64 (23)		169 (60)	62 (22)		
			250 (P)	Stable Coronary Heart Disease (100)	0	67 ± 9.2	28 (11)	14 (6)	69 (28)			61 (24)		138 (55)	39 (16)		
COLCOT [37]	RCT	2019	2366 (T)	STEMI (NA), NSTEMI (NA)	39 (1.6)	60.6 ± 10.7	472 (19.9)	708/2366 (29.9)	462 (19.5)	1185 (50.1)		370 (15.6)	48 (2.0)	392 (16.6)	69 (2.9)		
			2379 (P)	STEMI (NA), NSTEMI (NA)	50 (2.1)	60.5 ± 10.6	437 (18.4)	708/2377 (29.8)	497 (20.9)	1236 (52.0)		397 (16.7)	42 (1.8)	406 (17.1)	81 (3.4)		
SOLID TIMI 52 [38]	RCT	2014	6504 (T)	STEMI (46.1), NSTEMI (41.6) and UA (12.2)	43 (0.7)	64 (59-70)	1657 (25.5)	1227/6501 (18.9)	2275 (35.0)	4793 (73.7)	4191 (64.5)	2013 (31.0)		1532 (23.6)			
			6522 (P)	STEMI (44.2), NSTEMI (43.7), UA (12.1)	41 (0.6)	64 (59-71)	1669 (25.6)	1245/6513 (19.1)	2227 (34.1)	4762 (73.0)	4165 (63.9)	2033 (31.2)		1580 (24.2)			
STABILITY [39]	RCT	2014	7924 (T)	Stable Coronary Heart Disease (100)	80 (1.0)	65.0 (59.0–71.0)	1461 (18.4)	1572 (19.8)	2664 (33.6)			4681 (59.1)		3987 (50.3)	2644 (33.4)		
			7904 (P)	Stable Coronary Heart Disease (100)	69 (0.9)	65.0 (59.0–71.0)	1506 (19.1)	1656 (21.0)	2687 (34.0)			4642 (58.7)		3978 (50.3)	2592 (32.8)		
FRANCIS [41]	RCT	2010	313 (T)	STEMI (42.2), NSTEMI (36.4), UA (21.4)	6 (1.9)	58.5 ± 10.3^∗^	26.5	73 (23.3)	84 (26.8)	271 (86.6)	120 (38.3)			97 (31.0)		12.0 (0–222)	5.22 (2.79–12.10)
			311 (P)	STEMI (40.8), NSTEMI (33.4), UA (25.7)	3 (1.0)	59.6 ± 10.5^∗^	24.1	68 (21.9)	87 (28.0)	274 (88.1)	109 (35.0)			66 (21.2)		9.9 (0–377)	5.16 (2.69–10.94)
VISTA-16 [40]	RCT	2014	2572 (T)	STEMI (47.4), NSTEMI (37.4), UA (15.3)	26 (1.0)	61.0 (10.0)^∗^	691 (26.9)	854 (33.4)	801 (31.3)	1911 (75.2)	1255 (49.3)	769 (30.2)		453 (17.7)	161 (6.3)	11.4 (4.5-33.0)	
			2573 (P)	STEMI (46.9), NSTEMI (38.0), UA (15.1)	32 (1.2)	60.7 (9.8)^∗^	660 (25.7)	860 (33.6)	803 (31.3)	1977 (77.8)	1292 (50.9)	743 (29.6)		476 (18.6)	182 (7.1)	10.4 (4.0-28.7)	
CANTOS [17]	RCT	2017	2263 (T)	STEMI (53.6), NSTEMI (33.6), unknown type of missing data (12.8)	4 (0.2)	61.1 ± 10.1	606 (26.8)	536 (23.7)	888 (39.2)	1799 (79.5)			523 (23.1)	1509 (66.7)	316 (14)	4.15 (2.85-7.15)	2.59 (1.79-4.08)
			3344 (P)	STEMI (54.0), NSTEMI (33.9), unknown type of missing data (12.1)	9 (0.3)	61.1 ± 10	865 (25.9)	765 (22.9)	1333 (39.9)	2644 (79.1)			721 (21.6)	2192 (65.6)	469 (14)	41.1 (2.75-6.85)	2.61 (1.8-4.06)
SELECT-CABG [42]	RCT	2016	148 (T)	CABG (100)	NA	62.1 ± 9.2	16(10.8)										
			144 (P)	CABG (100)	NA	62.8 ± 8.2	15(10.4)										
LATITUDE TIMI-60 [43]	RCT	2016	1731 (T)	STEMI (25.0), NSTEMI (75.0)	NA	66 (61-74)	500 (28.9)	464 (26.8)	582 (33.6)	1268 (73.3)	985 (56.9)	425 (24.6)	206 (11.9)	412 (23.8)	154 (8.9)	3.6 (1.7-9.6)	
			1758 (P)	STEMI (24.6), NSTEMI (75.4)	NA	67 (61-73)	532 (30.3)	449 (25.6)	586 (33.3)	1276 (72.6)	936 (53.2)	426 (24.2)	216 (12.3)	410 (23.3)	137 (7.8)	3.7 (1.7-9.9)	
SOLSTICE 3	RCT	2012	192 (T)	NSTEMI (100)	3	63 (57–73)	57 (30)	56 (29)	59 (31)	136 (71)	115 (60)	46 (24)	16 (8)	55 (29)	21 (11)	37.1 (16.2-83.8)	5.3 (2.9-8.6)
			135 (P)	NSTEMI (100)	0	64 (56–71)	40 (30)	46 (34)	37 (27)	97 (72)	74 (55)	33 (17)	6 (4)	21 (16)	11 (8)	33.3 (13.3-102.9)	4.8 (2.8-9.8)

Studies with subgroup analysis. ∗ denotes mean (SD); otherwise, they are number (percentage). Range values are median (interquartile range). SD: standard deviation; NA: not available; T: treatment group; P: placebo group.

**Table 2:** Patient population and clinical scenario among included studies.

Patient population	Study	Investigational drug
STEMI	APEX-MI [28]	Pexelizumab
	COMMA [16]	
	COMPLY [16]	
	VCU-ART3 [34]	Anakinra
NSTEMI	MRC-ILA Heart Study [35]	Anakinra
MI (STEMI and NSTEMI)	CANTOS [17]	Canakinumab
	LATITUDE TIMI 60 [43]	Losmapimod
	COLCOT [37]	Colchicine
ACS (STEMI, NSTEMI, or UA)	SOLID TIMI 52 [38]	Darapladib
	FRANCIS [41]	Varespladib
	VISTA-16 [40]	Varespladib
CABG	PRIMO CABG 1 [32]	Pexelizumab
	PRIMO CABG 2 [31]	
	Pexelizumab Study Investigators [30]	
	SELECT-CABG [42]	Inclacumab
Stable CAD	STABILITY [39]	Darapladib
	LoDoCo [36]	Colchicine

^∗^Study with subgroup analysis of its primary end points.

**Table 3:** Subgroup analyses.

Clinical presentation					

	Stable CAD and CABG OR (CI)	STEMI OR (CI)	*P* value	NSTEMI OR (CI)	*P* value

All-cause mortality	0.87 (0.71-1.07)	0.89 (0.59-1.34)	0.92	2.47 (0.47-13.08)	0.22
Stroke	0.97 (0.60-1.55)	1.06 (0.69-1.62)	0.78	2.90 (0.12-72.20)	0.51
Recurrent MI	0.94 (0.81-1.09)	1.20 (0.89-1.62)	0.15	4.309 (0.84-19.84)	0.06
Revascularization	0.93 (0.82-1.09)	—	—	1.060 (0.65-3.95)	0.78
MACCE	0.81 (0.65-1.01)	1.02 (0.91-1.13)	0.07	1.29 (0.94-1.77)	0.02
Timing after acute event					
	**≤**7 days		>7 days		
All-cause mortality	1.08 (0.82-1.41)		0.94 (0.87-1.02)		0.33
Cardiac mortality	1.12 (0.70-1.79)		0.92 (0.83-1.02)		0.42
Stroke	1.03 (0.70-1.50)		0.90 (0.70-1.16)		0.56
Recurrent MI	1.34 (0.97-1.86)		0.92 (0.86-0.99)^∗^		0.03
Revascularization	1.54 (0.70-3.36)		0.83 (0.71-0.98)^∗^		0.03
MACCE	1.08 (0.93-1.26)		0.91 (0.82-1.00)		0.06
Clinical presentation					
	ACS		Non-ACS		
All-cause mortality	0.99 (0.88-1.11)		0.87 (0.71-1.07)		0.28
Cardiac mortality	0.92 (0.81-1.04)		0.94 (0.80-1.10)		0.84
Stroke	0.93 (0.74-1.16)		0.97 (0.60-1.55)		0.88
Recurrent MI	1.08 (0.91-1.27)		0.88 (0.80-0.98)^∗^		0.04
Revascularization	0.83 (0.65-1.07)		0.93 (0.82-1.05)		0.42
MACCE	0.99 (0.91-1.08)		0.83 (0.68-1.01)		0.11

## Supplementary Materials

Supplementary Materials Supplemental Table 1: bias risk assessment of the studies. Supplemental Figure 1: forest plot for network meta-analysis comparing the relative efficacy of each anti-inflammatory medication on all-cause death. Use of pexelizumab is associated with lower risk of all-cause mortality in comparison with veraspladib (OR 0.62, CI 0.37–0.99). Supplemental Figure 2: forest plot for network meta-analysis comparing the relative efficacies of each anti-inflammatory medication on cardiovascular death. Supplemental Figure 3: forest plot for network meta-analysis of myocardial infarction. Uses of canakinumab, colchicine, darapladib, and pexelizumab were associated with lower risk of recurrent myocardial infarction in comparison with anakinra (OR 0.20, CI 0.04–0.79; OR 0.21, CI 0.04–0.83; OR 0.22, CI 0.04–0.81; and OR 0.22, CI 0.05–0.82, respectively). Supplemental Figure 4: forest plot for network meta-analysis of revascularization. Use of colchicine significantly reduced the risk of revascularization in comparison to both anakinra and darapladib (OR 0.31, CI 0.11–0.84 and OR 0.52, CI 0.29–0.93, respectively). Supplemental Figure 5: forest plot for network meta-analysis of stroke. Use of colchicine was associated with significant reduced risk of stroke events after myocardial infarction in comparison to several anti-inflammatory medications including: darapladib (OR 0.23, CI 0.07-0.57), pexelizumab (OR 0.23, CI 0.07-0.64), losmapimod (OR 0.25, CI 0.07-0.85), canakinumab (OR 0.30, CI 0.09-0.81), and veraspladib (OR 0.26, CI 0.07-0.97). Supplemental Figure 6: forest plot for network meta-analysis comparing the relative efficacy of each anti-inflammatory medication on major adverse cardiac and cerebrovascular events (MAACE). Colchicine use was associated with significantly lower risk of MACCE when compared to darapladib (OR 0.69, CI 0.44-0.98), losmapimod (OR 0.60, CI 0.37-0.93), anakinra (OR 0.28 CI 0.10–0.70), and varespladib (OR 0.53, CI 0.32-0.83). Both canakinumab and pexelizumab were associated with reduced risk of MACCE (OR 0.37, CI 0.13–0.95 and OR 0.39, CI 0.14–0.96, respectively). Supplemental Figure 7: network plot of treatments included in this network meta-analysis. Circles represent the intervention as a node in the network, lines represent direct comparisons using randomized clinical trials (RCTs), and the thickness of lines corresponds to the number of (RCTs) included in each comparison. Supplemental Figure 8: funnel plots of odds ratios and standard errors to assess the publication bias of all-cause mortality, cardiac mortality, revascularization, stroke, recurrent myocardial infarction, and major adverse cardiac and cerebrovascular events (MACCE). Supplemental Figure 9: consistency plot for all-cause mortality, cardiac mortality, revascularization, stroke, recurrent myocardial infarction, and major adverse cardiac and cerebrovascular events (MACCE).
