# Supplementary material for: Comparative Effectiveness of Anti-Inflammatory Drug Treatments in Coronary Heart Disease Patients: A Systematic Review and Network Meta-Analysis
Source: Mediators Inflamm. 2021 Jan 14;2021:5160728. doi: 10.1155/2021/5160728 (PMC7822705; doi:10.1155/2021/5160728)
Supplement: Supplementary Materials — Supplemental Table 1: bias risk assessment of the studies. Supplemental Figure 1: forest plot for network meta-analysis comparing the relative efficacy of each anti-inflammatory medication on all-cause death. Use of pexelizumab is associated with lower risk of all-cause mortality in comparison with veraspladib (OR 0.62, CI 0.37–0.99). Supplemental Figure 2: forest plot for network meta-analysis comparing the relative efficacies of each anti-inflammatory medication on cardiovascular death. Supplemental Figure 3: forest plot for network meta-analysis of myocardial infarction. Uses of canakinumab, colchicine, darapladib, and pexelizumab were associated with lower risk of recurrent myocardial infarction in comparison with anakinra (OR 0.20, CI 0.04–0.79; OR 0.21, CI 0.04–0.83; OR 0.22, CI 0.04–0.81; and OR 0.22, CI 0.05–0.82, respectively). Supplemental Figure 4: forest plot for network meta-analysis of revascularization. Use of colchicine significantly reduced the risk of revascularization in comparison to both anakinra and darapladib (OR 0.31, CI 0.11–0.84 and OR 0.52, CI 0.29–0.93, respectively). Supplemental Figure 5: forest plot for network meta-analysis of stroke. Use of colchicine was associated with significant reduced risk of stroke events after myocardial infarction in comparison to several anti-inflammatory medications including: darapladib (OR 0.23, CI 0.07-0.57), pexelizumab (OR 0.23, CI 0.07-0.64), losmapimod (OR 0.25, CI 0.07-0.85), canakinumab (OR 0.30, CI 0.09-0.81), and veraspladib (OR 0.26, CI 0.07-0.97). Supplemental Figure 6: forest plot for network meta-analysis comparing the relative efficacy of each anti-inflammatory medication on major adverse cardiac and cerebrovascular events (MAACE). Colchicine use was associated with significantly lower risk of MACCE when compared to darapladib (OR 0.69, CI 0.44-0.98), losmapimod (OR 0.60, CI 0.37-0.93), anakinra (OR 0.28 CI 0.10–0.70), and varespladib (OR 0.53, CI 0.32-0.83). Both canakinumab and pexelizuma [file 5160728.f1.zip › Anti-inflammatory medication meta-analysis-Suppl Figures-FINAL.pdf]

Supplemental figure legends.

Supplemental Figure 1. Forest plot for network meta-analysis comparing the relative efficacy of each anti-inflammatory medication on all-cause death. Use of Pexelizumab is associated with lower risk of all- cause mortality in comparison with Veraspladib (OR 0.62, CI 0.37 – 0.99).

Supplemental Figure 2. Forest plot for network meta-analysis comparing the relative efficacies of each anti-inflammatory medication on cardiovascular death.

Supplemental Figure 3. Forest plot for network meta-analysis of myocardial infarction. Use of Canakinumab, Colchicine, Darapladib, and Pexelizumab were associated with lower risk of recurrent myocardial infarction in comparison with Anakinra (OR 0.20, CI 0.04 – 0.79; OR 0.21, CI 0.04 – 0.83; OR 0.22, CI 0.04 – 0.81; and OR 0.22, CI 0.05 – 0.82, respectively).

Supplemental Figure 4. Forest plot for network meta-analysis of revascularization. Use of Colchicine significantly reduced the risk of revascularization in comparison to both Anakinra and Darapladib (OR 0.31, CI 0.11 – 0.84; and OR 0.52, CI 0.29 – 0.93, respectively).

Supplemental Figure 5. Forest plot for network meta-analysis of stroke. Use of Colchicine was associated with significant reduced risk of stroke events after myocardial infarction in comparison to several anti-inflammatory medications including: Darapladib (OR 0.23, CI 0.07-0.57), Pexelizumab (OR 0.23, CI 0.07- 0.64), Losmapimod (OR 0.25, CI 0.07-0.85), Canakinumab (OR 0.30, CI 0.09-0.81), and Veraspladib (OR 0.26, CI 0.07-0.97).

Supplemental Figure 6. Forest plot for network meta-analysis comparing the relative efficacy of each anti-inflammatory medication on major adverse cardiac and cerebrovascular events **(**MAACE). Colchicine use was associated with significantly lower risk of MACCE when compared to Darapladib (OR 0.69, CI 0.44-0.98), Losmapimod (OR 0.60, CI 0.37-0.93), Anakinra (OR 0.28 CI 0.10 – 0.70) and Varespladib (OR 0.53, CI

0.32-0.83). Both Canakinumab and Pexelizumab were associated with reduced risk of MACCE (OR 0.37, CI 0.13 – 0.95 and OR 0.39, CI 0.14 – 0.96; respectively).

Supplemental Figure 7. network plot of treatments included in this network meta-analysis. Circles represent the intervention as a node in the network, lines represent direct comparisons using randomized clinical trials (RCTs), and the thickness of lines corresponds to the number of (RCTs) included in each comparison.

Supplemental Figure 8. Funnel plots of odds ratios and standard errors to assess the publication bias of all-cause mortality, cardiac mortality, revascularization, stroke, recurrent myocardial infarction, and major adverse cardiac and cerebrovascular events (MACCE).

Supplemental Figure 9. Consistency plot for all-cause mortality, cardiac mortality, revascularization, stroke, recurrent myocardial infarction, and major adverse cardiac and cerebrovascular events (MACCE).

All-cause death

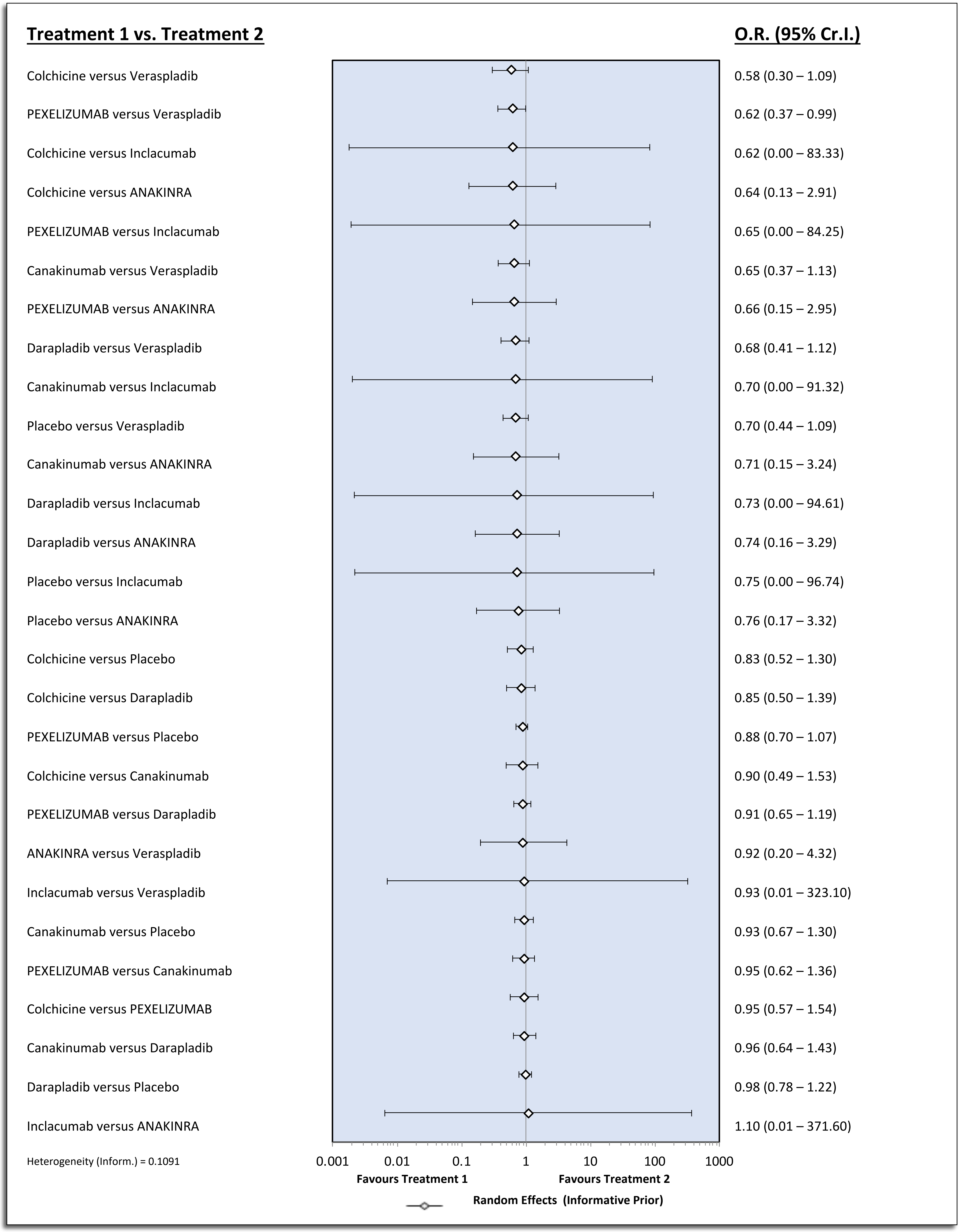

Supplemental Figure 1

All-cause death

**Colchicine**							
0.95 (0.57 – 1.54)	**PEXELIZUMAB**						
0.90 (0.49 – 1.53)	0.95 (0.62 – 1.36)	**Canakinumab**					
0.85 (0.50 – 1.39)	0.91 (0.65 – 1.19)	0.96 (0.64 – 1.43)	**Darapladib**				
0.83 (0.52 – 1.30)	0.88 (0.70 – 1.07)	0.93 (0.67 – 1.30)	0.98 (0.78 – 1.22)	**Placebo**			
0.62 (0.00 – 83.33)	0.65 (0.00 – 84.25)	0.70 (0.00 – 91.32)	0.73 (0.00 – 94.61)	0.75 (0.00 – 96.74)	**Inclacumab**		
0.64 (0.13 – 2.91)	0.66 (0.15 – 2.95)	0.71 (0.15 – 3.24)	0.74 (0.16 – 3.29)	0.76 (0.17 – 3.32)	1.10 (0.01 – 371.60)	**ANAKINRA**	
0.58 (0.30 – 1.09)	0.62 (0.37 – 0.99)	0.65 (0.37 – 1.13)	0.68 (0.41 – 1.12)	0.70 (0.44 – 1.09)	0.93 (0.01 – 323.10)	0.92 (0.20 – 4.32)	**Veraspladib**

Cardiovascular death

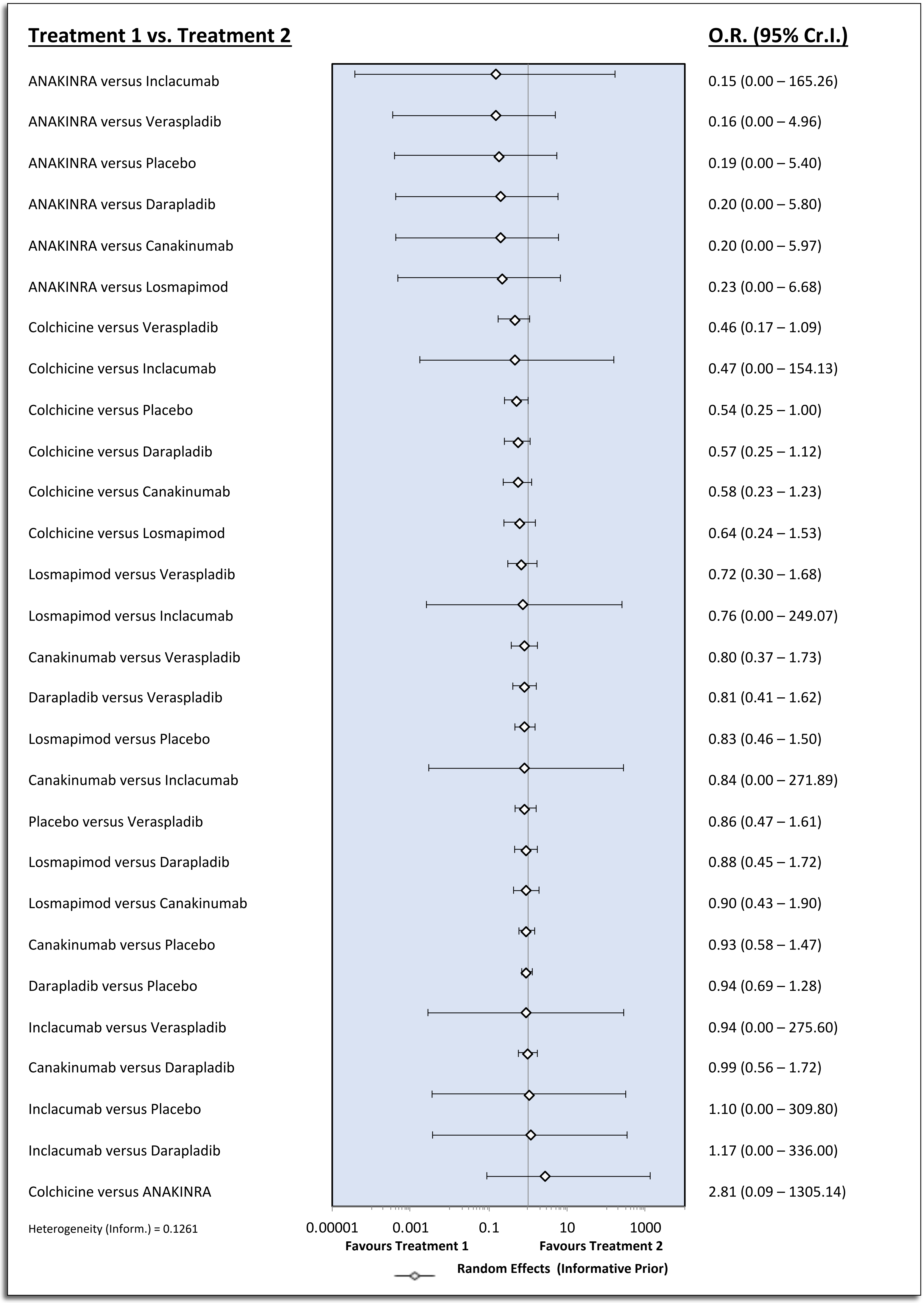

Supplemental Figure 2

Cardiovascular death

**Colchicine**							
2.81<br>(0.09 – 1305.14)	**ANAKINRA**						
0.64<br>(0.24 – 1.53)	0.23<br>(0.00 – 6.68)	**Losmapimod**					
0.58<br>(0.23 – 1.23)	0.20<br>(0.00 – 5.97)	0.90<br>(0.43 – 1.90)	**Canakinumab**				
0.47<br>(0.00 – 154.13)	0.15<br>(0.00 – 165.26)	0.76<br>(0.00 – 249.07)	0.84<br>(0.00 – 271.89)	**Inclacumab**			
0.57<br>(0.25 – 1.12)	0.20<br>(0.00 – 5.80)	0.88<br>(0.45 – 1.72)	0.99<br>(0.56 – 1.72)	1.17<br>(0.00 – 336.00)	**Darapladib**		
0.54<br>(0.25 – 1.00)	0.19<br>(0.00 – 5.40)	0.83<br>(0.46 – 1.50)	0.93<br>(0.58 – 1.47)	1.10<br>(0.00 – 309.80)	0.94<br>(0.69 – 1.28)	**Placebo**	
0.46<br>(0.17 – 1.09)	0.16<br>(0.00 – 4.96)	0.72<br>(0.30 – 1.68)	0.80<br>(0.37 – 1.73)	0.94<br>(0.00 – 275.60)	0.81<br>(0.41 – 1.62)	0.86<br>(0.47 – 1.61)	**Veraspladib**

MI

Treatment 1 vs. Treatment 2	O.R. (95% Cr.I.)
Canakinumab versus ANAKINRA	0.20 (0.04 – 0.79)
Colchicine versus ANAKINRA	0.21 (0.04 – 0.83)
Darapladib versus ANAKINRA	0.22 (0.04 – 0.81)
PEXELIZUMAB versus ANAKINRA	0.22 (0.05 – 0.82)
Placebo versus ANAKINRA	0.24 (0.05 – 0.86)
Inclacumab versus ANAKINRA	0.29 (0.04 – 2.17)
Losmapimod versus ANAKINRA	0.29 (0.06 – 1.14)
Veraspladib versus ANAKINRA	0.35 (0.07 – 1.39)
Canakinumab versus Veraspladib	0.58 (0.34 – 1.05)
Colchicine versus Veraspladib	0.61 (0.34 – 1.14)
Darapladib versus Veraspladib	0.62 (0.38 – 1.04)
PEXELIZUMAB versus Veraspladib	0.63 (0.39 – 1.05)
Placebo versus Veraspladib	0.67 (0.44 – 1.06)
Canakinumab versus Inclacumab	0.69 (0.15 – 2.95)
Canakinumab versus Losmapimod	0.71 (0.40 – 1.24)
Colchicine versus Inclacumab	0.72 (0.16 – 3.10)
Darapladib versus Inclacumab	0.73 (0.17 – 3.07)
Colchicine versus Losmapimod	0.74 (0.40 – 1.34)
PEXELIZUMAB versus Inclacumab	0.74 (0.17 – 3.11)
Darapladib versus Losmapimod	0.75 (0.46 – 1.23)
PEXELIZUMAB versus Losmapimod	0.76 (0.47 – 1.25)
Placebo versus Inclacumab	0.79 (0.18 – 3.25)
Placebo versus Losmapimod	0.81 (0.52 – 1.25)
Losmapimod versus Veraspladib	0.82 (0.45 – 1.56)
Inclacumab versus Veraspladib	0.85 (0.20 – 3.92)
Canakinumab versus Placebo	0.87 (0.61 – 1.24)
Colchicine versus Placebo	0.91 (0.60 – 1.38)
Canakinumab versus	0.92 (0.61 – 1.39)
Darapladib versus Placebo	0.93 (0.73 – 1.17)
Canakinumab versus Darapladib	0.93 (0.61 – 1.44)
PEXELIZUMAB versus Placebo	0.94 (0.76 – 1.17)
Canakinumab versus Colchicine	0.96 (0.56 – 1.66)
Colchicine versus PEXELIZUMAB	0.96 (0.60 – 1.53)
Colchicine versus Darapladib	0.98 (0.61 – 1.57)
Darapladib versus PEXELIZUMAB	0.99 (0.71 – 1.34)
Inclacumab versus Losmapimod	1.03 (0.23 – 4.72)

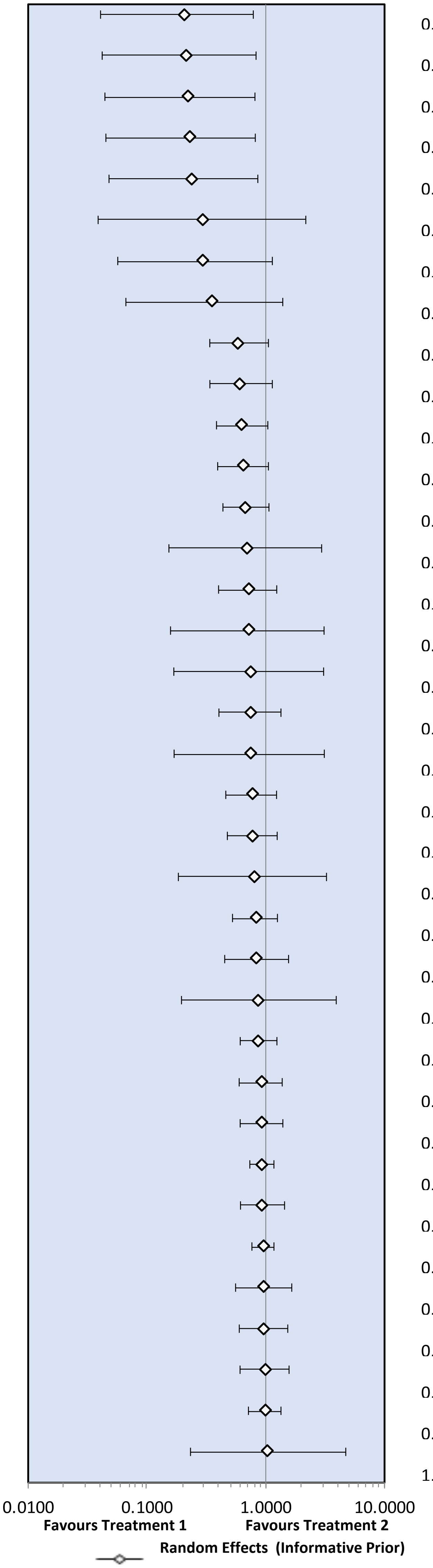

Heterogeneity (Inform.) = 0.1163

Supplemental Figure 3

MI

**Canakinumab**								
0.96<br>(0.56 – 1.66)	**Colchicine**							
0.93<br>(0.61 – 1.44)	0.98<br>(0.61 – 1.57)	**Darapladib**						
0.92<br>(0.61 – 1.39)	0.96<br>(0.60 – 1.53)	0.99<br>(0.71 – 1.34)	**PEXELIZUMAB**					
0.87<br>(0.61 – 1.24)	0.91<br>(0.60 – 1.38)	0.93<br>(0.73 – 1.17)	0.94<br>(0.76 – 1.17)	**Placebo**				
0.69<br>(0.15 – 2.95)	0.72<br>(0.16 – 3.10)	0.73<br>(0.17 – 3.07)	0.74<br>(0.17 – 3.11)	0.79<br>(0.18 – 3.25)	**Inclacumab**			
0.71<br>(0.40 – 1.24)	0.74<br>(0.40 – 1.34)	0.75<br>(0.46 – 1.23)	0.76<br>(0.47 – 1.25)	0.81<br>(0.52 – 1.25)	1.03<br>(0.23 – 4.72)	**Losmapimod**		
0.58<br>(0.34 – 1.05)	0.61<br>(0.34 – 1.14)	0.62<br>(0.38 – 1.04)	0.63<br>(0.39 – 1.05)	0.67<br>(0.44 – 1.06)	0.85<br>(0.20 – 3.92)	0.82<br>(0.45 – 1.56)	**Veraspladib**	
0.20<br>(0.04 – 0.79)	0.21<br>(0.04 – 0.83)	0.22<br>(0.04 – 0.81)	0.22<br>(0.05 – 0.82)	0.24<br>(0.05 – 0.86)	0.29<br>(0.04 – 2.17)	0.29<br>(0.06 – 1.14)	0.35<br>(0.07 – 1.39)	**ANAKINRA**

Revascularization

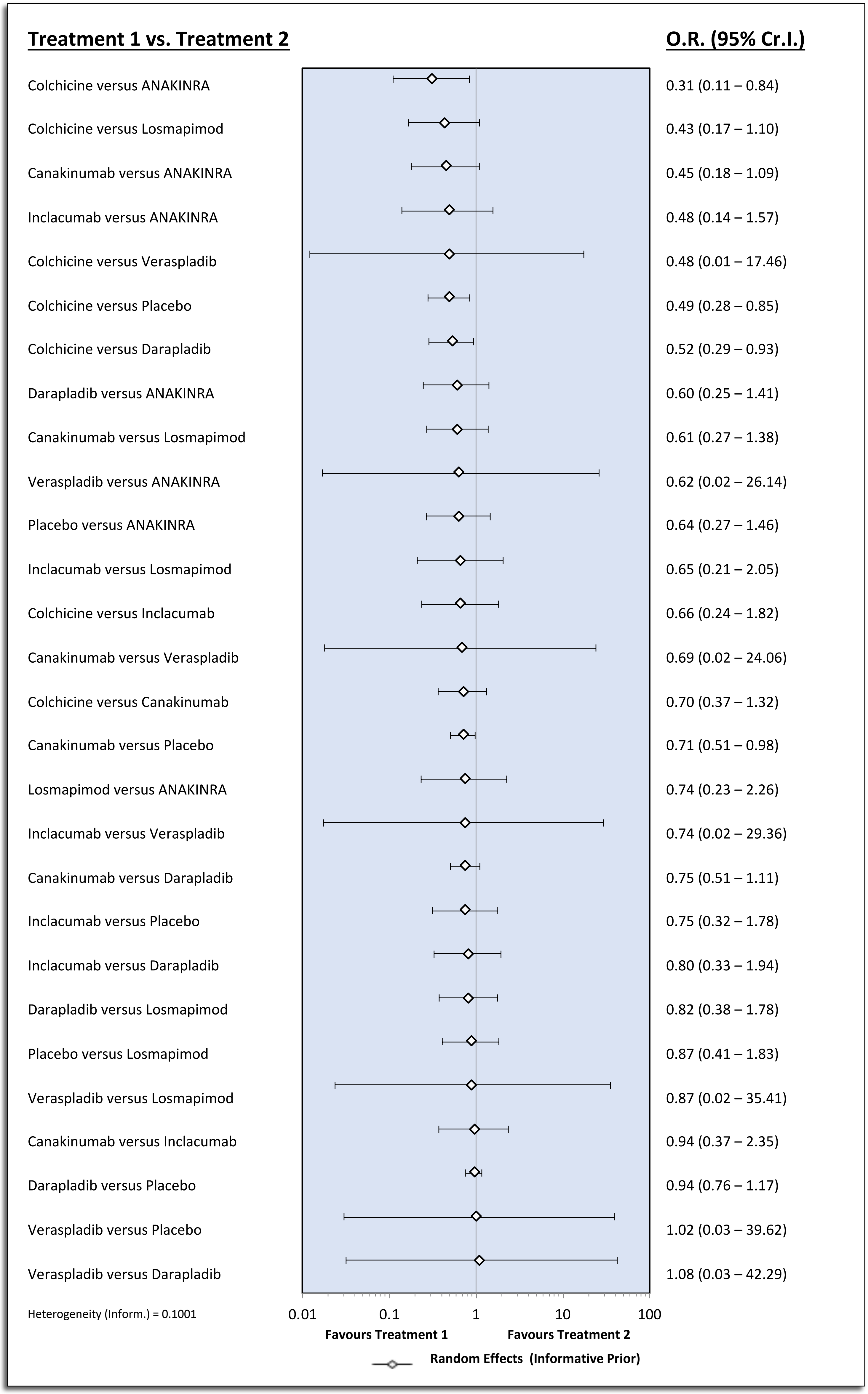

Supplemental Figure 4

Revascularization

**Colchicine**							
0.70 (0.37 – 1.32)	**Canakinumab**						
0.66 (0.24 – 1.82)	0.94 (0.37 – 2.35)	**Inclacumab**					
0.48 (0.01 – 17.46)	0.69 (0.02 – 24.06)	0.74 (0.02 – 29.36)	**Veraspladib**				
0.52 (0.29 – 0.93)	0.75 (0.51 – 1.11)	0.80 (0.33 – 1.94)	1.08 (0.03 – 42.29)	**Darapladib**			
0.49 (0.28 – 0.85)	0.71 (0.51 – 0.98)	0.75 (0.32 – 1.78)	1.02 (0.03 – 39.62)	0.94 (0.76 – 1.17)	**Placebo**		
0.43 (0.17 – 1.10)	0.61 (0.27 – 1.38)	0.65 (0.21 – 2.05)	0.87 (0.02 – 35.41)	0.82 (0.38 – 1.78)	0.87 (0.41 – 1.83)	**Losmapimod**	
0.31 (0.11 – 0.84)	0.45 (0.18 – 1.09)	0.48 (0.14 – 1.57)	0.62 (0.02 – 26.14)	0.60 (0.25 – 1.41)	0.64 (0.27 – 1.46)	0.74 (0.23 – 2.26)	**ANAKINRA**

Stroke

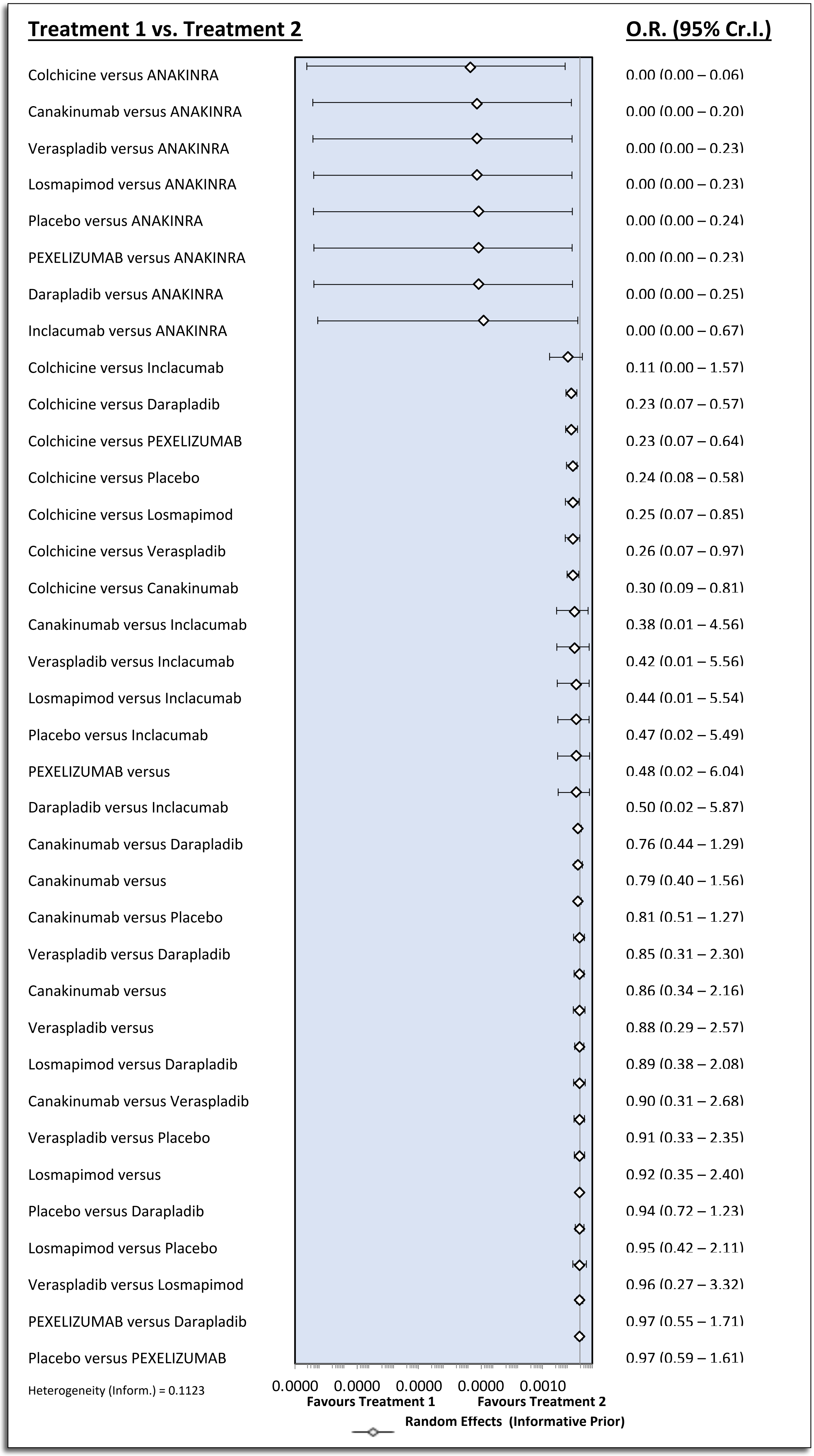

Supplemental Figure 5

Stroke

**Colchicine**								
0.30<br>(0.09 – 0.81)	**Canakinumab**							
0.26<br>(0.07 – 0.97)	0.90<br>(0.31 – 2.68)	**Veraspladib**						
0.25<br>(0.07 – 0.85)	0.86<br>(0.34 – 2.16)	0.96<br>(0.27 – 3.32)	**Losmapimod**					
0.24<br>(0.08 – 0.58)	0.81<br>(0.51 – 1.27)	0.91<br>(0.33 – 2.35)	0.95<br>(0.42 – 2.11)	**Placebo**				
0.23<br>(0.07 – 0.64)	0.79<br>(0.40 – 1.56)	0.88<br>(0.29 – 2.57)	0.92<br>(0.35 – 2.40)	0.97<br>(0.59 – 1.61)	**PEXELIZUMAB**			
0.23<br>(0.07 – 0.57)	0.76<br>(0.44 – 1.29)	0.85<br>(0.31 – 2.30)	0.89<br>(0.38 – 2.08)	0.94<br>(0.72 – 1.23)	0.97<br>(0.55 – 1.71)	**Darapladib**		
0.11<br>(0.00 – 1.57)	0.38<br>(0.01 – 4.56)	0.42<br>(0.01 – 5.56)	0.44<br>(0.01 – 5.54)	0.47<br>(0.02 – 5.49)	0.48<br>(0.02 – 6.04)	0.50<br>(0.02 – 5.87)	**Inclacumab**	
0.00<br>(0.00 – 0.06)	0.00<br>(0.00 – 0.20)	0.00<br>(0.00 – 0.23)	0.00<br>(0.00 – 0.23)	0.00<br>(0.00 – 0.24)	0.00<br>(0.00 – 0.23)	0.00<br>(0.00 – 0.25)	0.00<br>(0.00 – 0.67)	**ANAKINRA**

MACCE

Treatment 1 vs. Treatment 2	O.R. (95% Cr.I.)
Colchicine versus ANAKINRA	0.28 (0.10 – 0.70)
Canakinumab versus ANAKINRA	0.37 (0.13 – 0.95)
PEXELIZUMAB versus ANAKINRA	0.39 (0.14 – 0.96)
Darapladib versus ANAKINRA	0.41 (0.15 – 1.02)
Placebo versus ANAKINRA	0.42 (0.16 – 1.03)
Inclacumab versus ANAKINRA	0.43 (0.13 – 1.35)
Losmapimod versus ANAKINRA	0.47 (0.16 – 1.21)
Veraspladib versus ANAKINRA	0.53 (0.18 – 1.36)
Colchicine versus Veraspladib	0.53 (0.32 – 0.83)
Colchicine versus Losmapimod	0.60 (0.37 – 0.93)
Colchicine versus Inclacumab	0.65 (0.29 – 1.42)
Colchicine versus Placebo	0.67 (0.46 – 0.90)
Colchicine versus Darapladib	0.69 (0.44 – 0.98)
Canakinumab versus Veraspladib	0.70 (0.45 – 1.13)
Colchicine versus PEXELIZUMAB	0.72 (0.48 – 1.02)
PEXELIZUMAB versus Veraspladib	0.74 (0.50 – 1.09)
Colchicine versus Canakinumab	0.76 (0.46 – 1.14)
Darapladib versus Veraspladib	0.77 (0.52 – 1.17)
Canakinumab versus Losmapimod	0.80 (0.51 – 1.25)
Placebo versus Veraspladib	0.80 (0.57 – 1.14)
Inclacumab versus Veraspladib	0.81 (0.37 – 1.81)
PEXELIZUMAB versus Losmapimod	0.83 (0.58 – 1.20)
Canakinumab versus Inclacumab	0.87 (0.39 – 1.89)
Darapladib versus Losmapimod	0.87 (0.60 – 1.30)
Canakinumab versus Placebo	0.88 (0.64 – 1.20)
Losmapimod versus Veraspladib	0.88 (0.56 – 1.43)
Placebo versus Losmapimod	0.90 (0.66 – 1.25)
PEXELIZUMAB versus Inclacumab	0.91 (0.43 – 1.88)
Canakinumab versus Darapladib	0.91 (0.62 – 1.32)
Inclacumab versus Losmapimod	0.92 (0.42 – 2.03)
PEXELIZUMAB versus Placebo	0.92 (0.77 – 1.08)
PEXELIZUMAB versus Darapladib	0.95 (0.72 – 1.24)
Darapladib versus Inclacumab	0.96 (0.45 – 2.02)
Canakinumab versus PEXELIZUMAB	0.96 (0.67 – 1.37)
Darapladib versus Placebo	0.97 (0.78 – 1.20)
Inclacumab versus Placebo	1.01 (0.49 – 2.09)

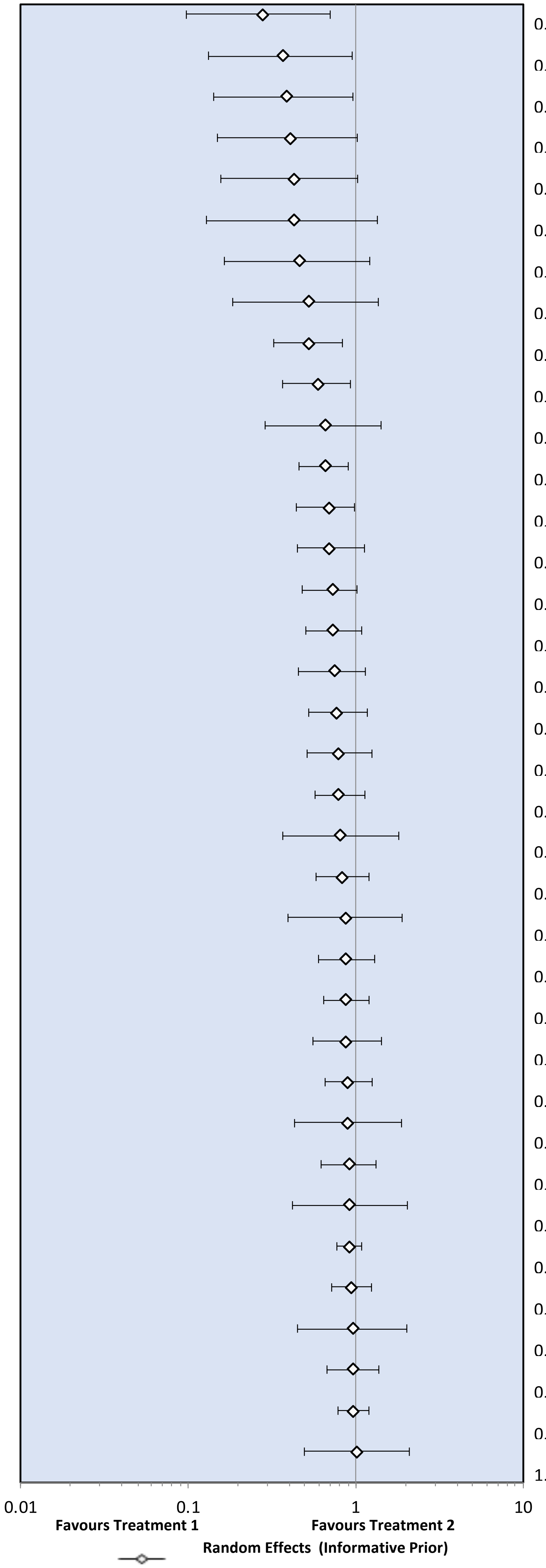

Heterogeneity (Inform.) = 0.1098

Supplemental Figure 6

MACCE

**Colchicine**								
0.76<br>(0.46 – 1.14)	**Canakinumab**							
0.72<br>(0.48 – 1.02)	0.96<br>(0.67 – 1.37)	**PEXELIZUMAB**						
0.69<br>(0.44 – 0.98)	0.91<br>(0.62 – 1.32)	0.95<br>(0.72 – 1.24)	**Darapladib**					
0.65<br>(0.29 – 1.42)	0.87<br>(0.39 – 1.89)	0.91<br>(0.43 – 1.88)	0.96<br>(0.45 – 2.02)	**Inclacumab**				
0.67<br>(0.46 – 0.90)	0.88<br>(0.64 – 1.20)	0.92<br>(0.77 – 1.08)	0.97<br>(0.78 – 1.20)	1.01<br>(0.49 – 2.09)	**Placebo**			
0.60<br>(0.37 – 0.93)	0.80<br>(0.51 – 1.25)	0.83<br>(0.58 – 1.20)	0.87<br>(0.60 – 1.30)	0.92<br>(0.42 – 2.03)	0.90<br>(0.66 – 1.25)	**Losmapimod**		
0.53<br>(0.32 – 0.83)	0.70<br>(0.45 – 1.13)	0.74<br>(0.50 – 1.09)	0.77<br>(0.52 – 1.17)	0.81<br>(0.37 – 1.81)	0.80<br>(0.57 – 1.14)	0.88<br>(0.56 – 1.43)	**Veraspladib**	
0.28<br>(0.10 – 0.70)	0.37<br>(0.13 – 0.95)	0.39<br>(0.14 – 0.96)	0.41<br>(0.15 – 1.02)	0.43<br>(0.13 – 1.35)	0.42<br>(0.16 – 1.03)	0.47<br>(0.16 – 1.21)	0.53<br>(0.18 – 1.36)	**ANAKINRA**

Network plot

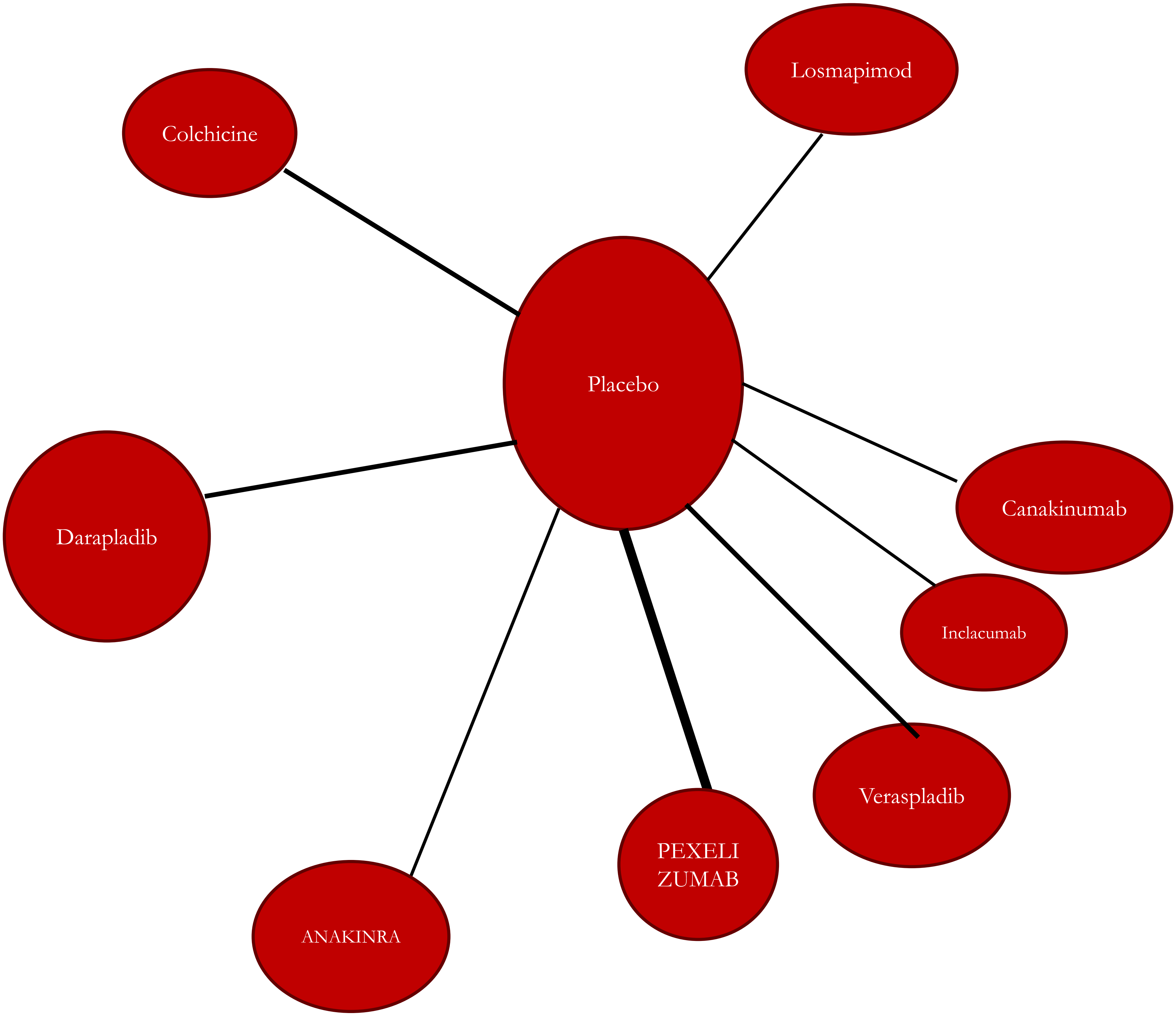

Supplemental Figure 7

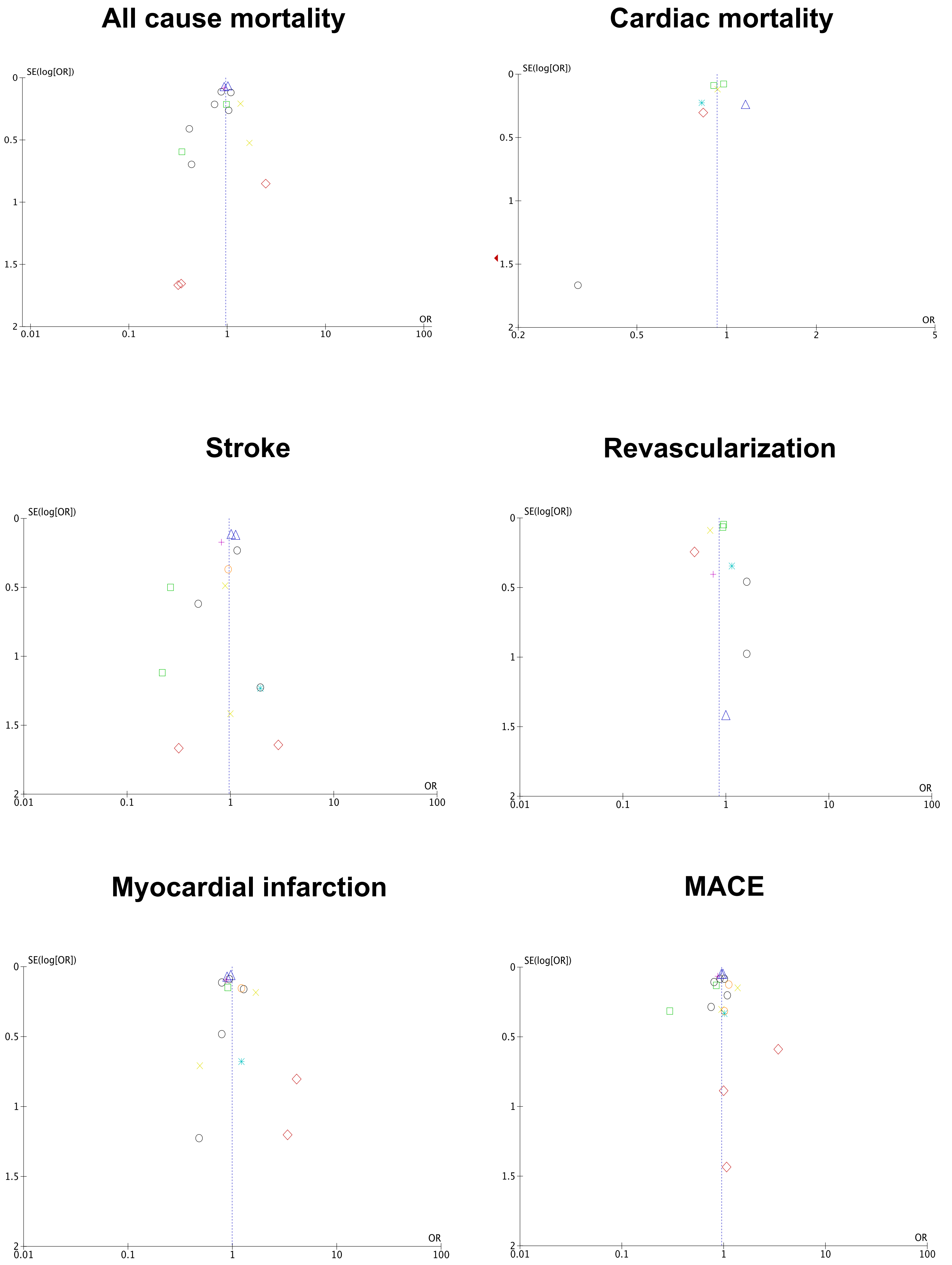

Supplemental Figure 8

Inconsistency analysis

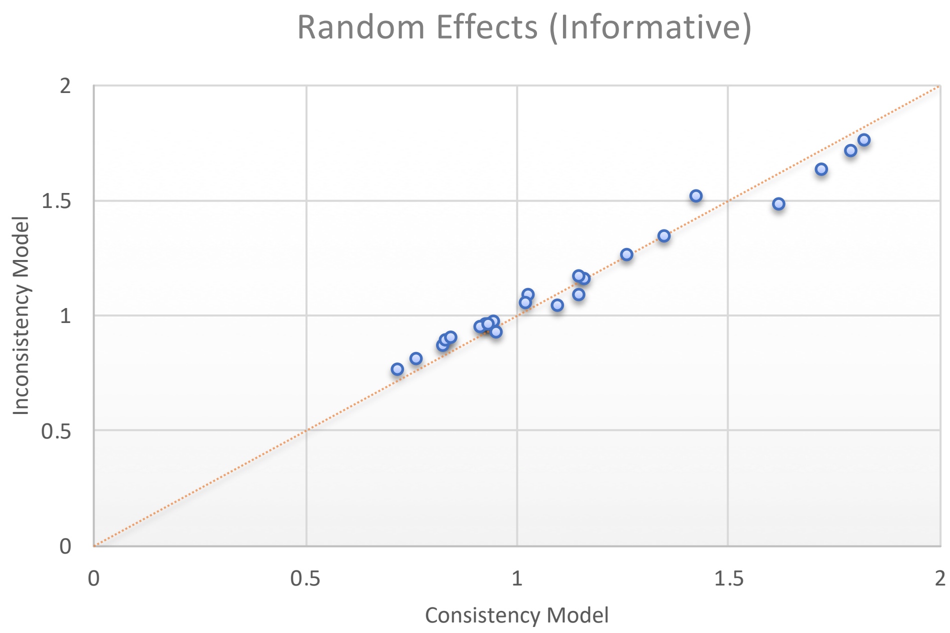

All-cause death

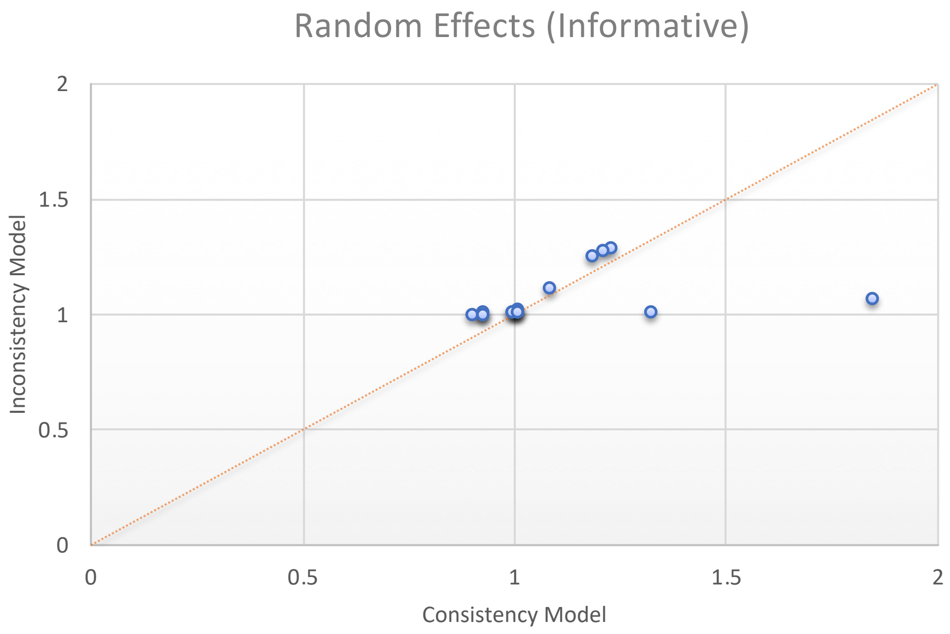

Cardiovascular death

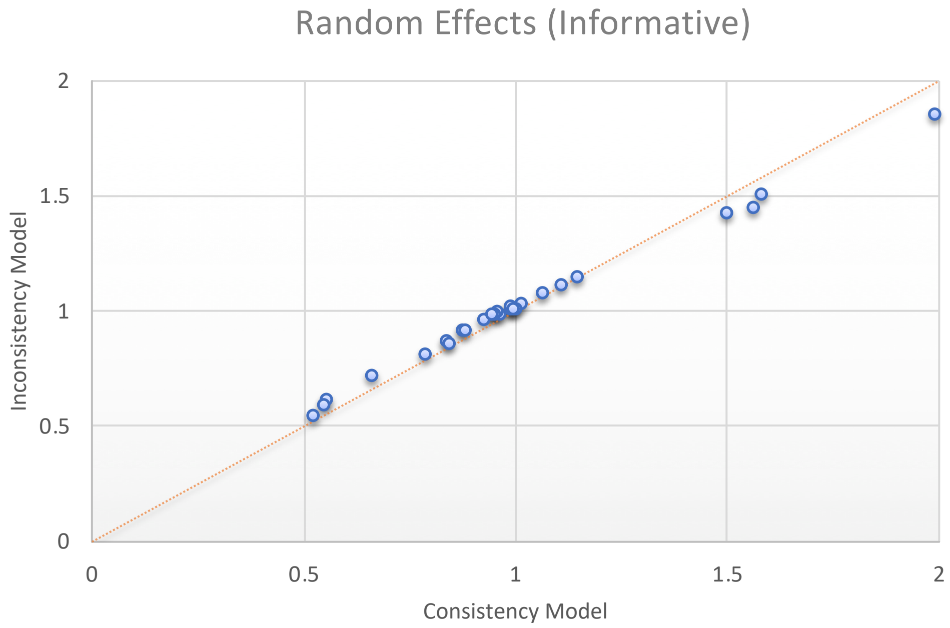

MI

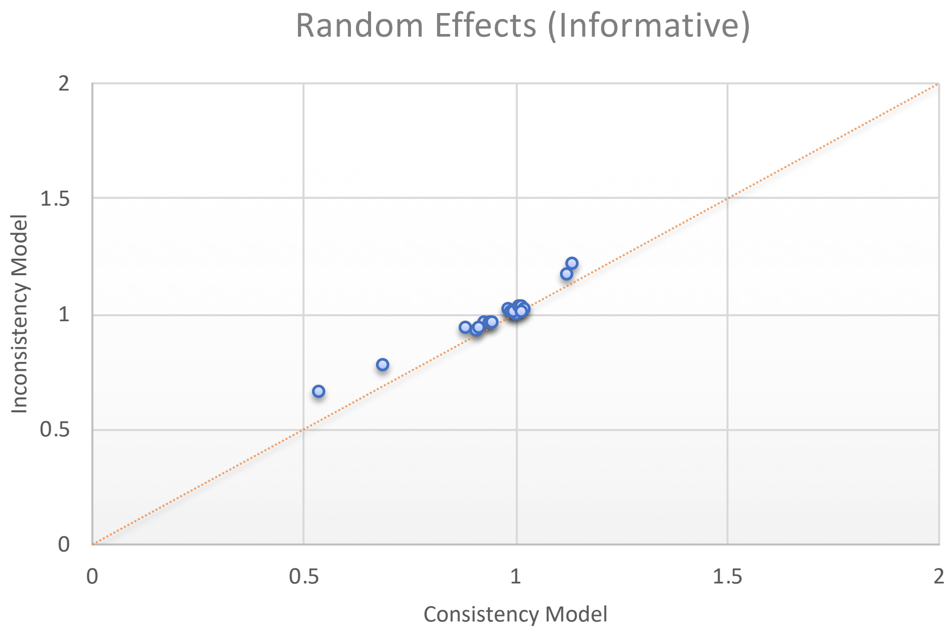

Revascularization

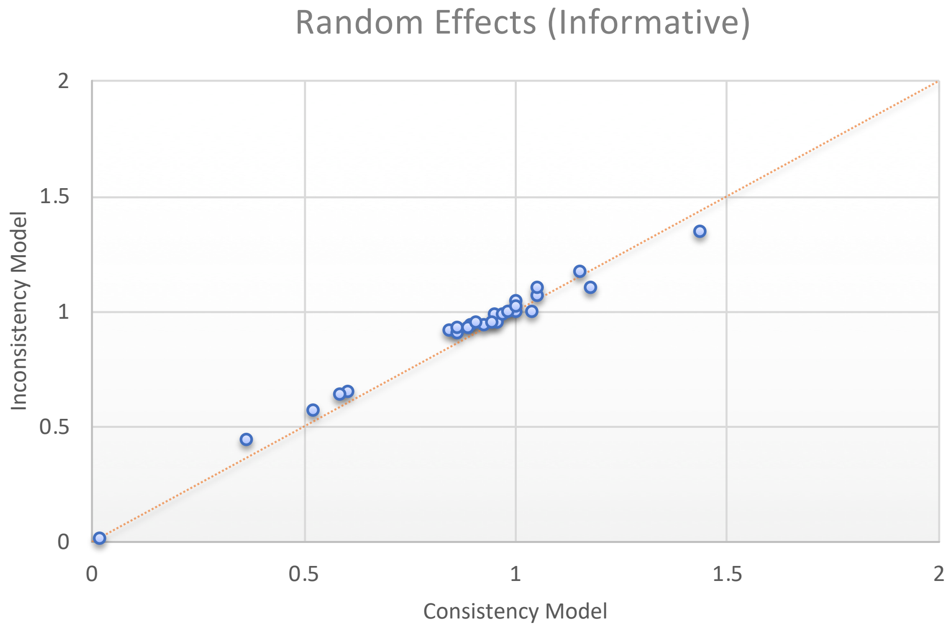

Stroke

Supplemental Figure 9
