# Supplementary material for: Comparative Effectiveness of Anti-Inflammatory Drug Treatments in Coronary Heart Disease Patients: A Systematic Review and Network Meta-Analysis
Source: Mediators Inflamm. 2021 Jan 14;2021:5160728. doi: 10.1155/2021/5160728 (PMC7822705; doi:10.1155/2021/5160728)
Supplement: Supplementary Materials — Supplemental Table 1: bias risk assessment of the studies. Supplemental Figure 1: forest plot for network meta-analysis comparing the relative efficacy of each anti-inflammatory medication on all-cause death. Use of pexelizumab is associated with lower risk of all-cause mortality in comparison with veraspladib (OR 0.62, CI 0.37–0.99). Supplemental Figure 2: forest plot for network meta-analysis comparing the relative efficacies of each anti-inflammatory medication on cardiovascular death. Supplemental Figure 3: forest plot for network meta-analysis of myocardial infarction. Uses of canakinumab, colchicine, darapladib, and pexelizumab were associated with lower risk of recurrent myocardial infarction in comparison with anakinra (OR 0.20, CI 0.04–0.79; OR 0.21, CI 0.04–0.83; OR 0.22, CI 0.04–0.81; and OR 0.22, CI 0.05–0.82, respectively). Supplemental Figure 4: forest plot for network meta-analysis of revascularization. Use of colchicine significantly reduced the risk of revascularization in comparison to both anakinra and darapladib (OR 0.31, CI 0.11–0.84 and OR 0.52, CI 0.29–0.93, respectively). Supplemental Figure 5: forest plot for network meta-analysis of stroke. Use of colchicine was associated with significant reduced risk of stroke events after myocardial infarction in comparison to several anti-inflammatory medications including: darapladib (OR 0.23, CI 0.07-0.57), pexelizumab (OR 0.23, CI 0.07-0.64), losmapimod (OR 0.25, CI 0.07-0.85), canakinumab (OR 0.30, CI 0.09-0.81), and veraspladib (OR 0.26, CI 0.07-0.97). Supplemental Figure 6: forest plot for network meta-analysis comparing the relative efficacy of each anti-inflammatory medication on major adverse cardiac and cerebrovascular events (MAACE). Colchicine use was associated with significantly lower risk of MACCE when compared to darapladib (OR 0.69, CI 0.44-0.98), losmapimod (OR 0.60, CI 0.37-0.93), anakinra (OR 0.28 CI 0.10–0.70), and varespladib (OR 0.53, CI 0.32-0.83). Both canakinumab and pexelizuma [file 5160728.f1.zip › Anti-inflammatory medication meta-analysis-Suppl Table 1-FINAL.pdf]

Supplemental Table 1. Bias risk assessment of the studies

Study name	Random sequence generation	Allocation concealment	Blind participants and personnel	Blind outcome assessment	Incomplete outcome data	Selective reporting
APEX-MI	Low risk	Low risk	Low risk	Low risk	Low risk	*Unclear* risk
COMPLY	Low risk	Low risk	Low risk	Low risk	Low risk	Low risk
COMMA	Low risk	Low risk	Low risk	Low risk	Low risk	Low risk
Pexelizumab Study Investigators	Low risk	Low risk	Low risk	Low risk	Low risk	Low risk
PRIMO CABG I	Low risk	Low risk	Low risk	Low risk	Low risk	Low risk
PRIMO CABG II	Low risk	Low risk	Low risk	Low risk	Low risk	Low risk
MRC-ILA Heart	Low risk	Low risk	Low risk	Low risk	Low risk	Low risk
VCU-ART I&II	Low risk	Low risk	Low risk	Low risk	Low risk	Low risk
VCU-ART3	Low risk	Low risk	Low risk	Low risk	Low risk	Low risk
COLCOT	Low risk	Low risk	Low risk	Low risk	Low risk	Low risk
LoDoCo	Low risk	Low risk	Low risk	Low risk	Low risk	Low risk
STABILITY	Low risk	Low risk	Low risk	Low risk	Low risk	Low risk
SOLID TIMI-52	Low risk	Low risk	Low risk	Low risk	Low risk	Low risk
FRANCIS	Low risk	Low risk	Low risk	Low risk	Low risk	Low risk
VISTA-16	Low risk	Low risk	Low risk	Low risk	*High* risk	Low risk
CANTOS	Low risk	Low risk	Low risk	Low risk	Low risk	Low risk
SELECT-CABG	Low risk	Low risk	Low risk	Low risk	*High* risk	*Unclear* risk
LATITUDE MI-60	Low risk	Low risk	Low risk	Low risk	Low risk	Low risk
SOLSTICE	Low risk	Low risk	Low risk	Low risk	Low risk	Low risk
